# Thrombomodulin reduces α-synuclein generation and ameliorates neuropathology in a mouse model of Parkinson’s disease

**DOI:** 10.1038/s41420-024-01939-y

**Published:** 2024-04-08

**Authors:** Xiao-yun Niu, Xi-xiu Xie, Hou-zhen Tuo, Cui-ping Lv, Ya-ru Huang, Jie Zhu, Shi-yu Liang, Xiao-yu Du, Cheng-gang Yang, Sheng-jie Hou, Xiao-ying Sun, Ling-jie Li, Fang Cui, Qi-xin Huang, Ying-bo Jia, Yu-jiong Wang, Rui-tian Liu

**Affiliations:** 1 College of Life Science, Ningxia University, Yinchuan, Ningxia China; 2 National Key Laboratory of Biochemical Engineering, Institute of Process Engineering, Chinese Academy of Sciences, Beijing, China; 3 Department of Neurology, Beijing Friendship Hospital, Capital Medical University, Beijing, China; 4 Department of BigData, Beijing Medintell Bioinformatic Technology Co., LTD, Beijing, China

**Keywords:** Parkinson's disease, Cellular neuroscience, Cell death in the nervous system

## Abstract

The neurotoxic α-synuclein (α-syn) oligomers play an important role in the occurrence and development of Parkinson’s disease (PD), but the factors affecting α-syn generation and neurotoxicity remain unclear. We here first found that thrombomodulin (TM) significantly decreased in the plasma of PD patients and brains of A53T α-syn mice, and the increased TM in primary neurons reduced α-syn generation by inhibiting transcription factor p-c-jun production through Erk1/2 signaling pathway. Moreover, TM decreased α-syn neurotoxicity by reducing the levels of oxidative stress and inhibiting PAR1-p53-Bax signaling pathway. In contrast, TM downregulation increased the expression and neurotoxicity of α-syn in primary neurons. When TM plasmids were specifically delivered to neurons in the brains of A53T α-syn mice by adeno-associated virus (AAV), TM significantly reduced α-syn expression and deposition, and ameliorated the neuronal apoptosis, oxidative stress, gliosis and motor deficits in the mouse models, whereas TM knockdown exacerbated these neuropathology and motor dysfunction. Our present findings demonstrate that TM plays a neuroprotective role in PD pathology and symptoms, and it could be a novel therapeutic target in efforts to combat PD.

**Figure:**
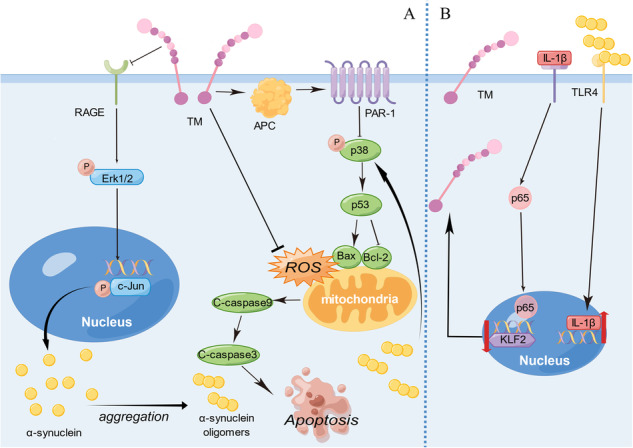
**Schematic representation of signaling pathways of TM involved in the expression and neurotoxicity of α-syn. A** TM decreased RAGE, and resulting in the lowered production of p-Erk1/2 and p-c-Jun, and finally reduce α-syn generation. α-syn oligomers which formed from monomers increase the expression of p-p38, p53, C-caspase9, C-caspase3 and Bax, decrease the level of Bcl-2, cause mitochondrial damage and lead to oxidative stress, thus inducing neuronal apoptosis. TM can reduce intracellular oxidative stress and inhibit p53-Bax signaling by activating APC and PAR-1. **B** The binding of α-syn oligomers to TLR4 may induce the expression of IL-1β, which is subsequently secreted into the extracellular space. This secreted IL-1β then binds to its receptor, prompting p65 to translocate from the cytoplasm into the nucleus. This translocation downregulates the expression of KLF2, ultimately leading to the suppression of TM expression. By Figdraw.

**Schematic representation of signaling pathways of TM involved in the expression and neurotoxicity of α-syn. A** TM decreased RAGE, and resulting in the lowered production of p-Erk1/2 and p-c-Jun, and finally reduce α-syn generation. α-syn oligomers which formed from monomers increase the expression of p-p38, p53, C-caspase9, C-caspase3 and Bax, decrease the level of Bcl-2, cause mitochondrial damage and lead to oxidative stress, thus inducing neuronal apoptosis. TM can reduce intracellular oxidative stress and inhibit p53-Bax signaling by activating APC and PAR-1. **B** The binding of α-syn oligomers to TLR4 may induce the expression of IL-1β, which is subsequently secreted into the extracellular space. This secreted IL-1β then binds to its receptor, prompting p65 to translocate from the cytoplasm into the nucleus. This translocation downregulates the expression of KLF2, ultimately leading to the suppression of TM expression. By Figdraw.

## Introduction

Parkinson’s disease (PD) is the second most common neurodegenerative disease [1]. It is characterized by the loss of dopamine neurons in the substantia nigra in the midbrain, and the presence of lewy bodies and lewy neurites composed of abnormally folded and aggregated α-synuclein (α-syn) [2, 3]. An imbalance between α-syn generation and clearance leads to α-syn accumulation and aggregation within the brains of PD patients. α-syn generation is influenced by a variety of factors including genetic, epigenetic, transcription factors and post-transcriptional modifications. Genetic mutation in SNCA (encoding for α-syn), such as duplication or triplication, cause increased transcription of SNCA mRNA and raised α-syn levels in the brain [4]. Previous reports indicated that Erk signaling pathway was involved in α-syn transcriptional activation [5].

α-syn monomers can aggregate to form oligomers, protofibrils and fibrils, and α-syn oligomers are considered as a major risk factor for neuronal deterioration in PD. Accumulation of α-syn inside mitochondria leads to mitochondrial complex I deficits, which induces the release of reactive oxygen species (ROS) and decreases the level of antioxidants, such as glutathione (GSH), superoxide dismutase (SOD), and glutathione peroxidase (GSH-Px). Oxidative damage further induces α-syn aggregation and impairs proteasomal ubiquitination and degradation of proteins, resulting in the formation of Lewy body protein aggregates in PD, thus, forming a deleterious cycle. α-syn oligomers also interact with mitochondrial membrane to release reactive cytochrome c into the cytosol, then active caspase-9 and caspase-3, triggering mitochondria-mediated apoptosis [6]. Moreover, α-syn oligomers secreted by neurons activate microglia and astrocytes by interacting with glial toll-like receptors (TLR), triggering gliosis and neuroinflammatory, therefore contributing to the death of dopaminergic neurons [7, 8].

Discovery and identification of the factors that regulate α-syn production, degradation and neurotoxicity have been a major objective in the PD field to better understand the pathological mechanism and develop effective therapeutics. Thrombomodulin (TM) is a glycosylated type I transmembrane molecule of 557 amino acids with multiple domains. Each domain possesses distinct properties. The molecule consists of an NH2-terminal lectin-like region followed by six tandem epidermal growth factor (EGF)-like structures, an O-glycosylation site-rich domain, a transmembrane domain, and a cytoplasmic tail domain. TM has been extensively investigated in regulating the processes of coagulation, and its novel functions are constantly reported [9, 10]. TM forms a complex with thrombin and protein C (PC), increasing PC activity. Activated PC (APC) binds to endothelial protein C receptor (EPCR), and activates protease-activated receptor 1 (PAR-1). PAR-1 activation downregulates the levels of the pro-apoptotic proteins such as p53 and Bax, while maintaining the levels of the anti-apoptotic protein Bcl-2 [11, 12]. Additionally, APC inhibits the activation of initiator caspases (e.g., caspase-8) and effector caspases (e.g., caspase-3) and suppresses oxidative stress, thus exerting anti-apoptotic effects [11]. Previous reports have indicated that the loss of TM function in multiple sclerosis (MS) results in the functional impairment of APC. This impairment subsequently leads to mitochondrial dysfunction and increased oxidative stress within the nervous system [13]. Injecting recombinant human soluble TM (rsTM), which is composed of extra-membrane domain of TM, has been shown to reduce mitochondrial damage and the production of oxidative stress [13]. Moreover, rsTM has been approved for the treatment of disseminated intravascular coagulation in Japan in 2008 [14, 15]. However, the role of q2TM in PD pathogenesis remains elusive. In this study, we first analyzed the levels of TM in the PD patients and A53T mice, and then investigated its effect on α-syn generation and neurotoxicity in vitro and in vivo.

## Results

### Decreased expression of TM in PD patients and PD mouse models

**Fig. 1:**
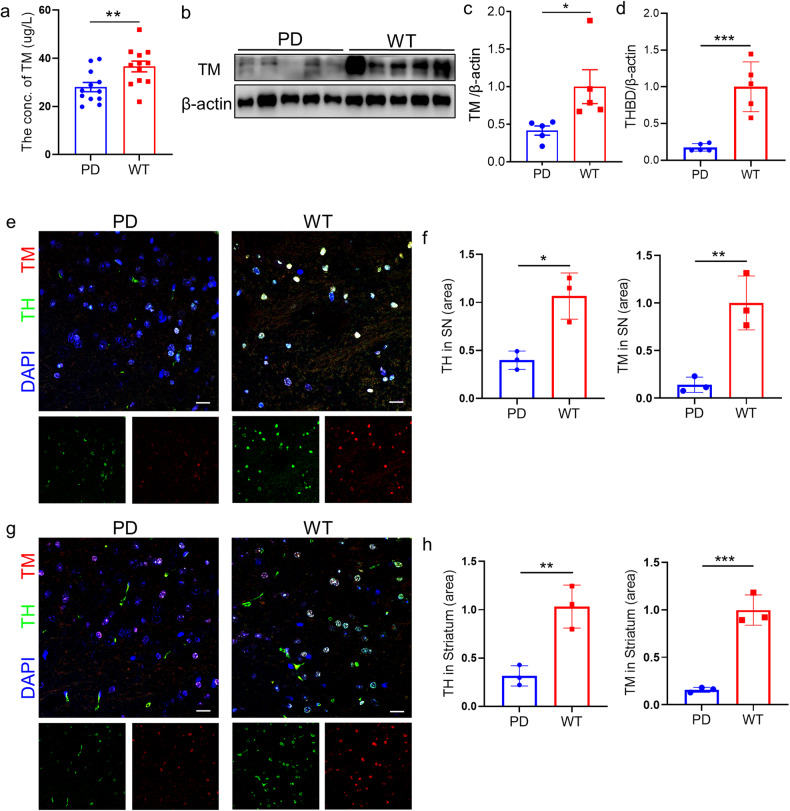
The levels of TM significantly decreased in the brains of A53T α-syn mice. **a** TM levels in the plasma of A53T α-syn mice. TM concentrations in the plasma of A53T α-syn mice (*n* = 12) and WT mice (*n* = 12) were detected by ELISA kit. Data are mean ± SEM, and an unpaired *t* test with two-tailed was used for statistical analysis. **b** The levels of TM in the brain homogenates of A53T α-syn mice and wild-type (WT) mice were detected by western blotting (*n* = 5 mice per group). **c** The densitometry analysis of TM in (**b**). Data are mean ± SEM, and an unpaired *t* test with two-tailed was used for statistical analysis. **d** The relative mRNA levels of THBD (encodes TM) in the brain homogenates of A53T α-syn mice and WT mice were analyzed by qPCR (*n* = 5 mice per group). Data are mean ± SEM, and an unpaired *t* test with two-tailed was used for statistical analysis. **e**, **g** Representative confocal images of TH (Green) and TM (red) in the substantia nigra (**e**) or striatum (**g**) of A53T α-syn mice and WT mice. Scale bar represents 20 μm. **f**, **h** The fluorescent area of TH and TM in the substantia nigra (**e**) or striatum (**g**) were quantified by IpWin32 software. *n* = 3 mice per group. Data are mean ± SEM, and an unpaired *t* test with two-tailed was used for statistical analysis. *P < 0.05, **P < 0.01, ***P < 0.001.

To investigate the possible factors affecting PD development, we analyzed the plasma levels of TM in patients with PD and A53T mice. The levels of TM significantly decreased in the plasma of PD patients and A53T mice compared with control (Supplementary Fig. S1a and Fig. 1a). Moreover, our western blotting results showed that TM protein levels also significantly decreased in the brains of 15-month-old A53T α-syn mice (Fig. 1b, c). Consistently, the mRNA levels of THBD were decreased in the brains of A53T α-syn mice (Fig. 1d). These results were further confirmed by comparing the fluorescence of TM in the substantia nigra and striatum of A53T α-syn mice with WT mice via IHC (Fig. 1e–h).

### TM reduces α-syn generation

**Fig. 2:**
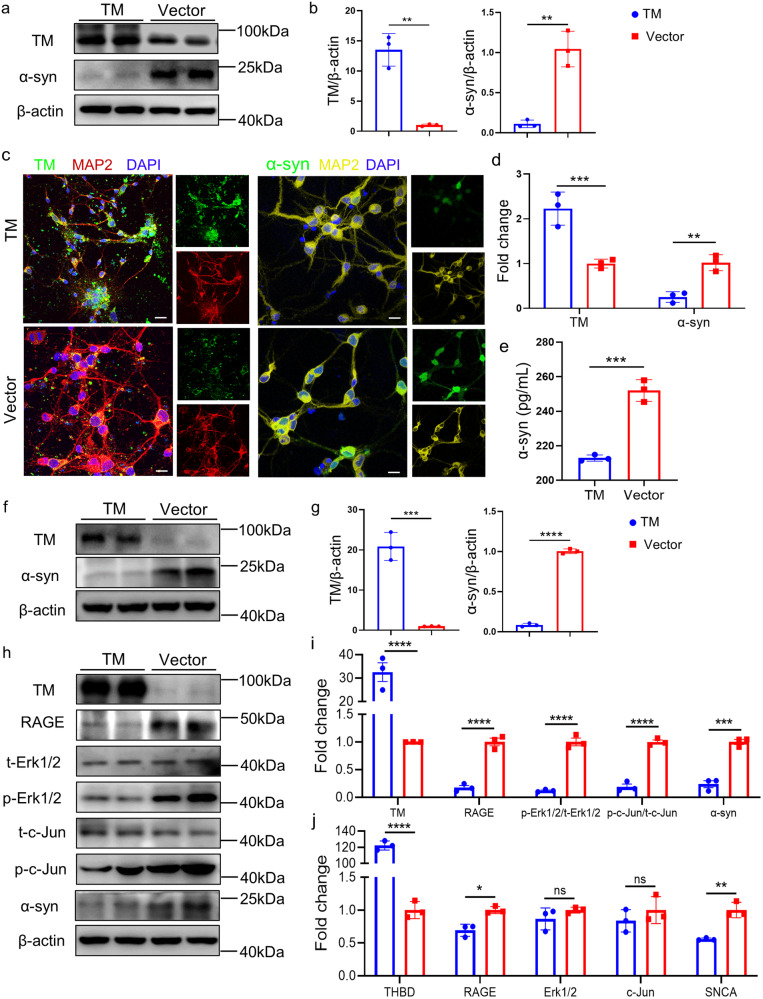
TM reduces α-syn generation in neurons. **a** Representative western blotting of TM and α-syn in the PC12 cells treated with TM plasmid (TM). **b** Densitometry analysis of TM and α-syn in (**a**). *n* = 3 represents three independent experiments. Data are mean ± SEM, and an unpaired *t* test with two-tailed was used for statistical analysis. **c** Representative confocal images of TM (green) and α-syn (green) in the primary neurons (DIV10, left panel: red; right panel: yellow) treated with lentiviral carrying TM plasmid or Vector plasmid for 72 h. Scale bar represents 10 μm. **d** The fluorescent area of TM and α-syn in (**c**) was quantified by IpWin32 software. *n* = 3 represents three independent experiments. Data are mean ± SEM, and an unpaired *t* test with two-tailed was used for statistical analysis. **e** The levels of α-syn in primary neurons (DIV10) treated with TM or Vector was determined by ELISA. *n* = 3 represents three independent experiments. Data are mean ± SEM, and an unpaired *t* test with two-tailed was used for statistical analysis. **f** Representative western blotting of TM and α-syn in the primary neurons treated with TM plasmid (TM). **g** Densitometry analysis of TM and α-syn in (**f**). *n* = 3 represents three independent experiments. Data are mean ± SEM, and an unpaired *t* test with two-tailed was used for statistical analysis. **h** The levels of TM, RAGE, t-Erk1/2, p-Erk1/2, t-c-Jun, p-c-Jun and α-syn in primary neurons with TM overexpression were analyzed by western blotting. β-actin was used as a control. **i** Relative levels of TM, RAGE, t-Erk1/2, p-Erk1/2, t-c-Jun, p-c-Jun and α-syn in (**h**) were quantified using Image J software. *n* = 3 represents three independent experiments. Data are mean ± SEM, and a one-way ANOVA followed by Tukey’s multiple comparison test. **j** The mRNA levels of THBD, RAGE, Erk1/2, c-Jun and SNCA (encodes α-syn) in primary neurons with TM overexpression or control were detected by qPCR. *n* = 3 represents three independent experiments. Data are mean ± SEM, and a one-way ANOVA followed by Tukey’s multiple comparison test. *P < 0.05, **P < 0.01, ***P < 0.001, ****P < 0.0001, ns, not significant.

To investigate the effect of TM on α-syn production, we detected the levels of α-syn in PC12 cells with human α-syn overexpression by western blotting. The results showed that overexpression of TM significantly decreased the levels of α-syn (Fig. 2a, b). By contrast, downregulation of TM markedly increased the α-syn levels (Supplementary Fig. S1b, c). Consistently, ICC, ELISA and WB results showed that upregulation of TM in primary neurons remarkably decreased the levels of α-syn compared with control group (Fig. 2c–g). By contrast, downregulation of TM markedly increased the α-syn levels (Supplementary Fig. S1d–h).

To determine whether the TM upregulation-induced α-syn decreases attributes to the decreased synthesis or promoted degradation of α-syn, CHX was added to the primary neurons to inhibit protein synthesis, and then α-syn protein levels were analyzed. As shown in Supplementary Fig. S1i, j, TM overexpression did not affect the degradation of α-syn.

### TM mediates α-syn expression through Erk signaling pathway

Previous reports demonstrate that advanced glycation end product (RAGE) promotes Erk1/2 phosphorylation (p-Erk1/2) which regulates the transcription of α-syn [16, 17]. Our present results showed that overexpression of TM in primary neurons remarkedly decreased the levels of RAGE, p-Erk1/2 and also p-c-Jun, preventing α-syn expression (Fig. 2h, i), whereas TM knock-down significantly increased their levels (Supplementary Fig. S2a, b). Consistently, our PCR results showed that TM mediated the corresponding transcription changes of α-syn by regulating RAGE transcription (Fig. 2j and Supplementary Fig. S2c). Moreover, 30 μM honokiol (HNK, an activator of p-Erk1/2) treatment significantly reversed the effects of TM overexpression in the levels of p-Erk1/2, p-c-Jun and α-syn, which further confirmed that TM regulated α-syn generation through Erk signaling pathway (Supplementary Fig. S2d–i). Our results also suggested that TM mediated the phosphorylation of Erk1/2 and c-Jun rather than their transcription. We also investigated the effect of TM on the levels of oligomeric and phosphorylated α-syn by western blotting, the results showed that TM significantly reduced α-syn aggregation and phosphorylation in primary neurons (Supplementary Fig. S3a–d).

### TM reduces α-syn oligomer-induced neurotoxicity by blocking apoptosis-related signaling pathways

**Fig. 3:**
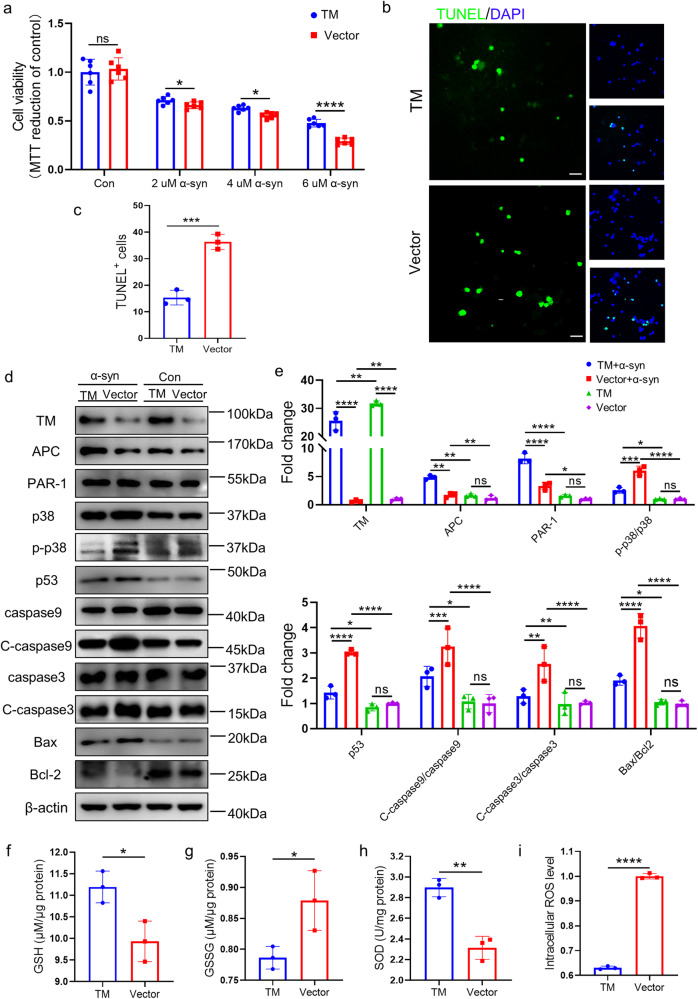
TM reduces α-syn-induced cell apoptosis and oxidative stress. **a** The effect of TM overexpression on the viability of primary neurons. Primary neurons (DIV8) with TM overexpression treated with different concentration of α-syn oligomers for 72 h, and then cells were detected by MTT assay. *n* = 6 represents technical replicates. Experiment was repeated at least three times. Data are mean ± SEM, and an unpaired *t* test with two-tailed was used for statistical analysis. **b** Primary neurons (DIV8) were treated with 2 μM α-syn oligomers for 72 h after infected with TM or control, then the cells were detected by TUNEL method. Scale bar represents 25 μm. **c** The fluorescent area of TUNEL^+^ cells in (**b**) was quantified by IpWin32 software. *n* = 3 represents three independent experiments. Data are mean ± SEM, and an unpaired *t* test with two-tailed was used for statistical analysis. **d** The effect of TM overexpression on the levels of TM, APC, RAR-1, p38, p-p38, p53, caspase9, Cleved-caspase9 (C-caspase9), caspase3, Cleved-caspase3 (C-caspase3), Bax and Bcl-2 in primary neurons after treated with 2 μM α-syn oligomers. β-actin was used as a control. **e** Relative levels of TM, APC, PAR-1, p-p38/p38, p53, C-caspase9/caspase9, C-caspase3/caspase3, Bax and Bcl-2 in (**d**) were quantified using Image J software, respectively. *n* = 3 represents three independent experiments. Data are mean ± SEM, and a one-way ANOVA followed by Tukey’s multiple comparison test. **f**–**i** The effect of TM overexpression on the levels of oxidative stress (GSH, GSSG, SOD and ROS). Primary neurons infected with TM or Vector were incubated with 2 μM α-syn oligomers for 48 h. Then the levels of oxidative stress were detected by ELISA kit. *n* = 3 represents three independent experiments. Data are mean ± SEM, and an unpaired *t* test with two-tailed was used for statistical analysis. *P < 0.05, **P < 0.01, ***P < 0.001, ****P < 0.0001, ns not significant.

To investigate the effect of TM on the neurotoxicity of α-syn oligomers, we added α-syn oligomers to primary neurons infected with TM. The results showed that compared with the control group, TM overexpression significantly enhanced the cell viability of primary neurons treated with different concentrations of α-syn oligomers (Fig. 3a). In contrast, downregulation of TM significantly increased α-syn oligomers-induced toxicity (Supplementary Fig. S4a). Consistently, TM overexpression decreased α-syn oligomers-induced neuronal apoptosis detected by TUNEL staining, whereas TM downregulation increased the apoptosis (Supplementary Figs. 3b, c and S 4b, c).

Previous reports showed that α-syn oligomers activated p38 signaling pathway, causing neuronal apoptosis [18], and TM activates PAR-1 through a complex composed of TM, thrombin and PC, while PAR-1 downregulates the levels of p38, p53 and Bax and maintains the levels of the anti-apoptotic protein Bcl-2 [11, 12]. Our present western blotting results showed that the levels of APC, PAR-1 were significantly increased, while phosphorylated p38 (p-p38)/ p38, p53, Cleaved-caspase9(C-cas9)/caspase9, Cleaved-caspase3(C-cas3)/C-caspase3 and Bax/Bcl-2 were obviously reduced in the primary neurons infected with TM relative to vector controls (Fig. 3d, e). In contrast, downregulation of TM significantly decreased the levels of APC, PAR-1, and increased (p-p38)/ p38, p53, C-cas9/caspase9, C-cas3/C-caspase3 and Bax/Bcl-2 (Supplementary Fig. S4d, e). Accordingly, our qPCR results indicated that TM mediated the transcription of APC, PAR-1and apoptosis-related protein such as p38, p53, C-caspase3 and Bax through opposite regulation (Supplementary Fig. S5a, b).

Oxidative stress plays an important role in the degeneration of dopaminergic neurons in PD [19]. We then assessed the levels of oxidative stress in the primary neurons with TM overexpression or knockdown. The levels of GSH and SOD were significantly increased in TM overexpression group, whereas the levels of GSSG and ROS were significantly decreased (Fig. 3f–i). In contrast, downregulation of TM reversed these changes (Supplementary Fig. S4f–i).

### α-syn oligomers prevent TM expression by inhibiting KLF2

To determine the association of TM expression with α-syn oligomers in A53T mice, we treated primary cortical neurons with α-syn oligomers, and found that α-syn oligomers markedly decreased TM levels. Previous report has shown that KLF2 acts as a transcription factor to regulate the expression of TM, which is inhibited by IL-1β. IL-1β binds to its receptor, resulting in the translocation of P65 from the cytoplasm into the nucleus [20]. Accordingly, KLF2 levels in primary cortical neurons treated with α-syn oligomers were detected. The results indicated that α-syn oligomers significantly increased IL-1β levels, prompted p65 to translocate from the cytoplasm into the nucleus, reduced KLF2 level and TM expression (Supplementary Fig. S6a–d).

### Neuronal TM overexpression attenuates motor and memory deficits in A53T α-syn mice

To determine the effect of TM on PD pathology and symptoms, AAV carrying TM gene (AAV-TM, Supplementary Fig. S7a) and TM shRNA (AAV-shTM, Supplementary Fig. S7b) were injected into the substantia nigra region of A53T α-syn mice (Supplementary Fig. S7c). We first examined the distribution of AAV expression by observing the mCherry-expressing cells in the mouse brains 30 days after injection. The mCherry^+^ cells widely distributed in brainstem region of the mice injected with AAV-TM and shTM, and co-located with neurons rather than astrocytes and microglia (Supplementary Fig. S7d). As expected, TM levels significantly increased in the neurons infected with AAV-TM (Supplementary Fig. S7e, f) or obviously decreased in the neurons infected with AAV-shTM (Supplementary Fig. S7g, h).

**Fig. 4:**
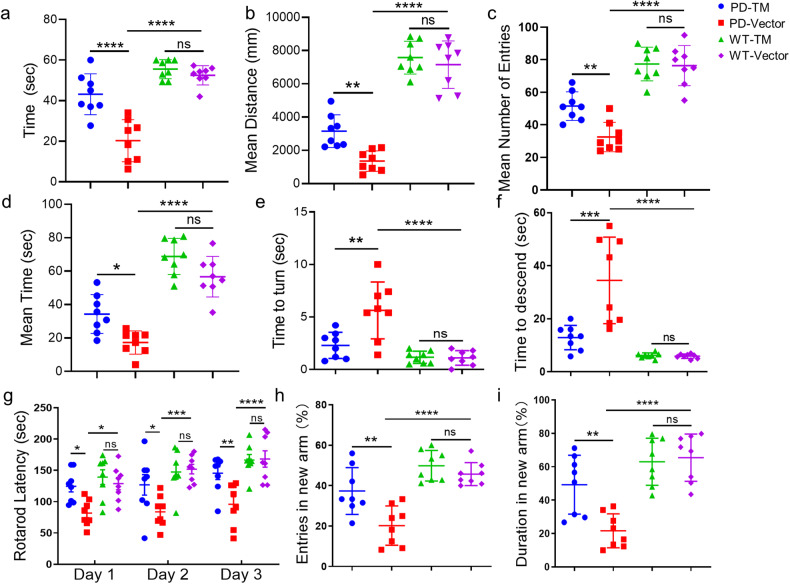
TM attenuates the motor deficits in A53T α-syn mice. The mice were stereotactically injected with AAV-TM or AAV-Vector, their motor behavioral and cognitive abilities were tested 30 days after injection. **a** Body suspension test was used to detect mouse muscle by recording the time in which the mice hold on the rung. *n* = 8 mice per group. Data are mean ± SEM, and a one-way ANOVA followed by Tukey’s multiple comparison test was used for statistical analysis. **b**–**d** The motor activity of mice was detected via the distance in center (**b**), times to entire center region (**c**) and time of mice (**d**) spent in center. *n* = 8 mice per group. Data are mean ± SEM, and a one-way ANOVA followed by Tukey’s multiple comparison test was used for statistical analysis. **e**, **f** The balance and coordination of mice detected by pole test. The time of mice with TM overexpression to turn (**e**) and descend (**f**) was recorded in this experiment. *n* = 8 mice per group. Data are mean ± SEM, and a one-way ANOVA followed by Tukey’s multiple comparison test was used for statistical analysis. **g** The exercise ability of mice was measured by the rotarod test, the latency to fall from rotarod during the test in 3 consecutive days. *n* = 8 mice per group. Data are mean ± SEM, and a one-way ANOVA followed by Tukey’s multiple comparison test was used for statistical analysis. **h**, **i** The special memory of mice detected by Y maze, the time spent in the novel arm (**h**) and the number of entries (**i**) in Y-maze test. *n* = 8 mice per group. Data are mean ± SEM, and a one-way ANOVA followed by Tukey’s multiple comparison test was used for statistical analysis. *P < 0.05, **P < 0.01, ***P < 0.001, ****P < 0.0001, ns not significant.

Muscle coordination and grip strength of fore- and hind-limbs were assessed by suspending the mice to a bar. The results revealed that WT mice were able to grasp the bar with the forelimbs for almost 60 s, but the hold time of vector-treated A53T mice was reduced to 20 s. Treatment with TM significantly increased the hold time to more than 40 s (Fig. 4a).

We then tested the effect of TM on the motor of A53T α-syn mice. The open field test results showed that TM-treated A53T α-syn mice exhibited higher locomotor activity than vector-treated mice by a higher number of times entering the entire center region, a longer distance and time traveled in the center region (Fig. 4b–d).

Impaired balance and coordination are the main symptoms of PD. Pole test and rotarod test were used to evaluate the effects of TM treatment on the balance and coordination of transgenic A53T mice. The pole test showed that A53T mice treated with TM took a shorter time to turn and descend from the pole compared with that treated with vector (Fig. 4e, f). Moreover, the rotarod test revealed that TM-treated mice markedly enhanced the rotarod latency of mice (Fig. 4g).

Y-maze tests were conducted to evaluate the effect of TM on cognitive function in A53T mice. The results showed that TM-treated mice spent more time in the new arm (Fig. 4h) and exhibited more times in entering into the new arm than the vehicle-treated mice (Fig. 4i). However, upregulation of TM did not induce significant effect on WT mice (Fig. 4a–i). By contrast, downregulation of TM lowered motor activity, impaired the balance and coordination, and cognitive function of A53T mice (Supplementary Fig. S8a–i).

### TM reduces α-syn and increases DA levels in the brains of A53T mice

**Fig. 5:**
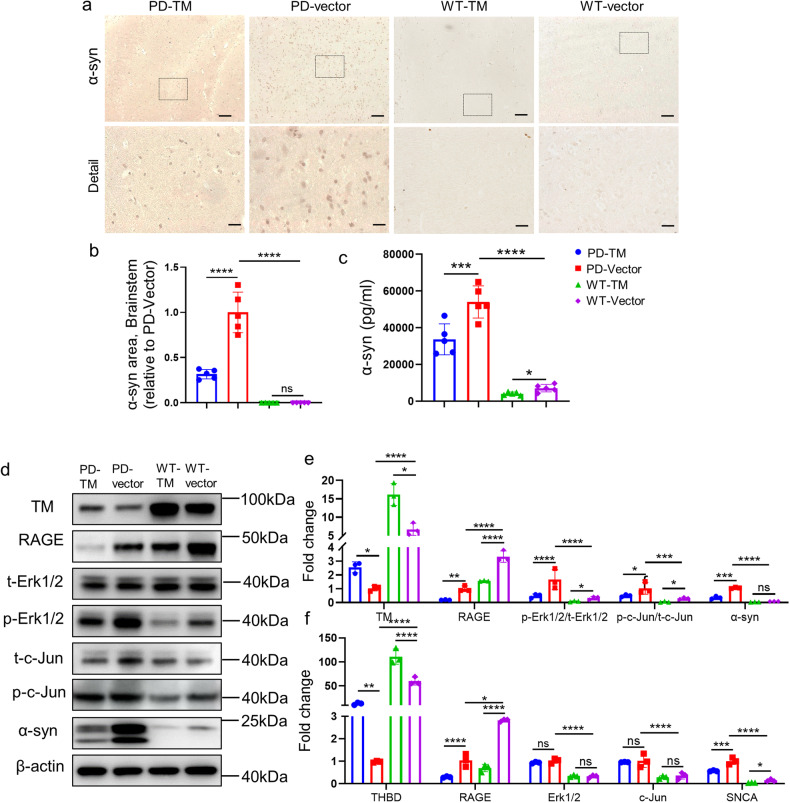
TM reduces the level of α-syn in the brains of A53T mice. **a** Representative images of α-syn in brainstem of mice treated with TM. Scale bar: 100 μm. **b** The area of α-syn in brainstem of mouse brains in (**a**) was quantified using IpWin32 software. n = 5 mice per group. Data are mean ± SEM, and a one-way ANOVA followed by Tukey’s multiple comparison test was used for statistical analysis. **c** α-syn in the brain homogenate of mice treated with TM were analyzed by ELISA kit. *n* = 5 mice per group. Data are mean ± SEM, and a one-way ANOVA followed by Tukey’s multiple comparison test was used for statistical analysis. **d** The levels of TM, RAGE, t-Erk1/2, p-Erk1/2, t-c-Jun, p-c-Jun and α-syn in the brainstem of A53T mice with TM overexpression were analyzed by western blotting. β-actin was used as a control. **e** Relative levels of TM, RAGE, t-Erk1/2, p-Erk1/2, t-c-Jun, p-c-Jun and α-syn in (**d**) were quantified using Image J software. *n* = 3 represents three independent experiments. Data are mean ± SEM, and a one-way ANOVA followed by Tukey’s multiple comparison test. **f** The mRNA levels of THBD, RAGE, Erk1/2, c-Jun and α-syn in the brainstem of mice with TM overexpression. *n* = 3 represents three independent experiments. Data are mean ± SEM, and a one-way ANOVA followed by Tukey’s multiple comparison test was used for statistical analysis. *P < 0.05, **P < 0.01, ***P < 0.001, ****P < 0.0001, ns not significant.

We further investigated the effect of TM on α-syn levels in A53T α-syn mice by IHC, ELISA, western blotting and qPCR. Our IHC results showed that TM-treated mice significantly decreased the levels of α-syn in the brainstem of A53T mice compared with vector-treated mice (Fig. 5a, b). The ELISA results consistently showed that α-syn levels significantly decreased in the brain homogenates of mice treated with AAV-TM relative to AAV-vector (Fig. 5c). Consistent with our in vitro results, AAV-TM also significantly reduced α-syn levels in A53T mice via the Erk signaling pathway (Fig. 5d, e). Moreover, our qPCR results showed that the transcription levels of related proteins in α-syn generation pathway were also significantly decreased (Fig. 5f). Downregulation of TM led to a marked enhancement of α-syn levels (Supplementary Fig. S9a–f).

**Fig. 6:**
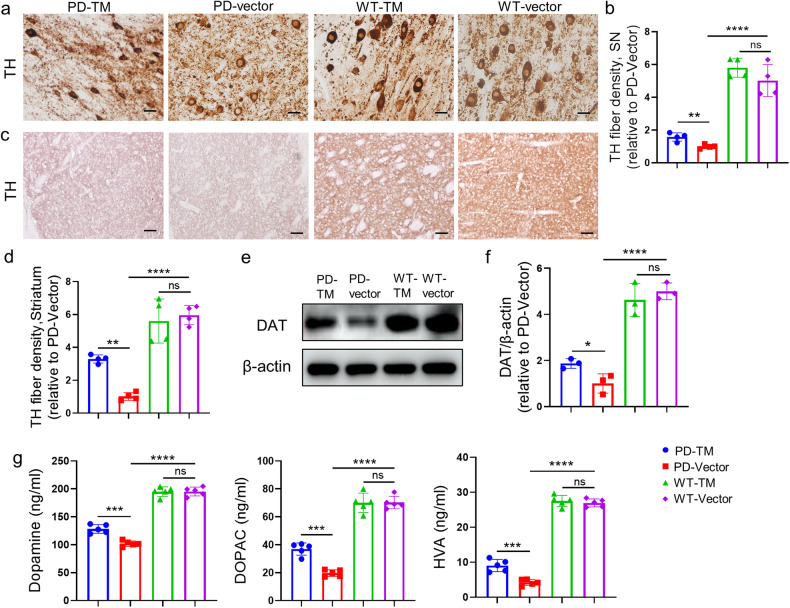
TM improves the level of dopamine in the brains of A53T mice. **a** The levels of TH in substantia nigra of mice with TM overexpression were detected by immunohistochemistry with anti-TH antibody. Scale bar: 20 μm. **b** Quantification of TH fiber densities in (**a**) using IpWin32 software. *n* = 4 mice per group. Data are mean ± SEM, and a one-way ANOVA followed by Tukey’s multiple comparison test was used for statistical analysis. **c** The levels of TH in striatum of mice with TM overexpression were detected by IHC. Scale bar: 100 μm. **d** Quantification of TH fiber densities in (**c**) using IpWin32 software. *n* = 4 mice per group. Data are mean ± SEM, and a one-way ANOVA followed by Tukey’s multiple comparison test was used for statistical analysis. **e** The levels of DAT in substantia nigra of A53T mice with TM overexpression were analyzed by western blotting. β-actin was used as a control. **f** Densitometry analysis of DAT in (**e**). *n* = 3 represents three independent experiments. Data are mean ± SEM, and an unpaired *t* test with two-tailed was used for statistical analysis. **g** DA and its metabolites DOPAC, HVA in the brainstem of mice treated with or without TM were analyzed by ELISA kit. *n* = 5 mice per group. Data are mean ± SEM, and a one-way ANOVA followed by Tukey’s multiple comparison test was used for statistical analysis. *P < 0.05, **P < 0.01, ***P < 0.001, ****P < 0.0001, ns, not significant.

The effect of TM on DA pathology in A53T mice was detected by ELISA and IHC analysis. It is well known that TH catalyzes the formation of dopamine precursor (L-DOPA), which is a rate-limiting step in the biosynthesis of DA. Our results showed that TH levels in the substantia nigra (Fig. 6a, b) and striatum (Fig. 6c, d) of TM-treated mouse group were significantly increased compared with that of control. Similarly, western blotting results showed that the levels of dopamine transporter (DAT) were significantly increased in the substantia nigra of TM-treated A53T mouse group (Fig. 6e, f), and ELISA results further showed that the levels of DA and its downstream metabolites HVA and DOPAC were significantly higher than those in the control group (Fig. 6g). Downregulation of TM resulted in decreases in TH, DA, HVA and DOPAC levels (Supplementary Fig. S10a–g).

### TM attenuates neuronal apoptosis in A53T α-syn mice

**Fig. 7:**
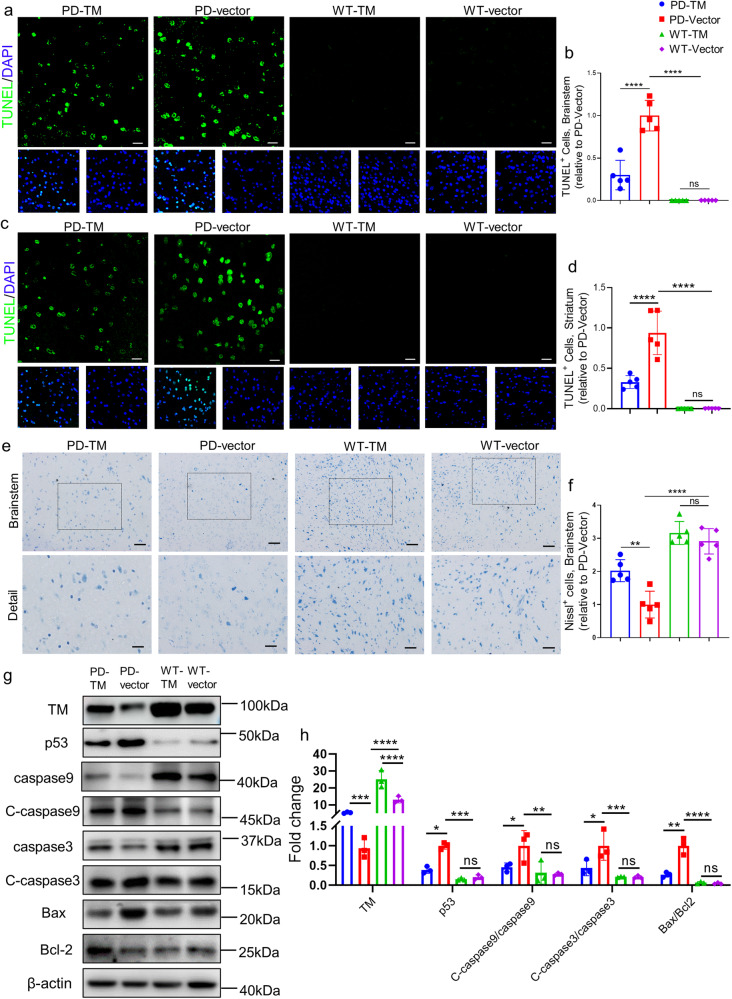
TM reduces the levels of neuropathology and α-syn in the brains of A53T α-syn mice. **a** Representative images of TUNEL (green) and DAPI (blue) in brainstem of TM-treated mice. Scale bar represents 25 μm. **b** Quantification of TUNEL^+^ cells in the brainstem regions in (**a**). *n* = 5 mice per group. Data are mean ± SEM, and a one-way ANOVA followed by Tukey’s multiple comparison test was used for statistical analysis. **c** Representative images of TUNEL (green) and DAPI (blue) in striatum of TM-treated mice. Scale bar represents 25 μm. **d** Quantification of TUNEL^+^ cells in the striatum regions in (**c**). *n* = 5 mice per group. Data are mean ± SEM, and a one-way ANOVA followed by Tukey’s multiple comparison test was used for statistical analysis. **e** Representative images of Nissl staining in brainstem of TM-treated mice. Scale bar represents 100 μm. Detail scale bar: 50 μm. **f** Quantification of Nissl^+^ cells in brainstem regions in (**e**) using IpWin32 software. *n* = 5 mice per group. Data are mean ± SEM, and a one-way ANOVA followed by Tukey’s multiple comparison test was used for statistical analysis. **g** TM and apoptosis-related proteins in TM-treated mouse brain homogenate were analyzed by western blotting. **h** Relative levels of TM, p53, Caspase9, C-caspase9, Caspase3, C-caspase3, Bax, Bcl-2 in (**g**) were quantified using Image J software. *n* = 3 represents three independent experiments. Data are mean ± SEM, and a one-way ANOVA followed by Tukey’s multiple comparison test was used for statistical analysis. *P < 0.05, **P < 0.01, ***P < 0.001, ****P < 0.0001, ns not significant.

We next applied TUNEL staining to reveal the effect of TM on neuronal apoptosis in A53T α-syn mice, the results showed that abundant TUNEL-positive cells were found in brainstem and striatum of A53T mice, while AAV-TM treatment significantly decreased the number of TUNEL-positive cells (Fig. 7a–d). By contrast, TUNEL-positive cells were significantly increased in the shTM-treated group (Supplementary Fig. S11a–d). Further examination by Nissl staining to assess the number of neurons in brainstem and striatum. We found that neuronal TM upregulation significantly increased the number of nissl body in the brainstem and striatum of A53T mice injected with AAV-TM while TM reduction decreased the number of neurons, indicating that TM distinctly ameliorated neuronal damage (Fig. 7e, f and Supplementary Fig. S12a–f). No significant difference was observed between TM-treated and vector-treated WT mice. Consistently, our western blotting results revealed that the levels of apoptosis-related proteins including p53, C-caspase9, C-caspase3 and Bax/Bcl-2 were significantly reduced in the brains of TM-treated A53T α-syn mice (Fig. 7g, h), while TM knockdown increased their levels (Supplementary Fig. S12g, h).

### TM attenuates oxidative stress and neuroinflammation

**Fig. 8:**
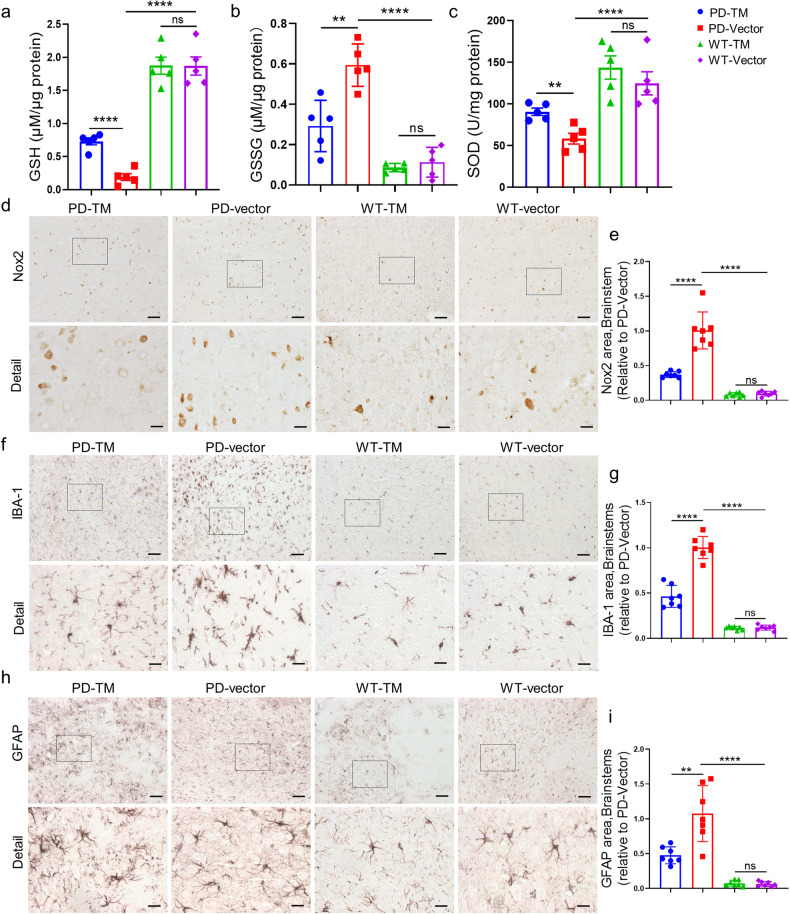
TM reduces the levels of oxidative stress, attenuates gliosis and neuroinflammation in the brains of A53T α-syn mice. **a**–**c** Oxidative stress related indexes GSH (**a**), GSSG (**b**) and SOD (**c**) in striatum of mice treated with TM were analyzed by oxidative stress detection kit. *n* = 5 mice per group. Data are mean ± SEM, and a one-way ANOVA followed by Tukey’s multiple comparison test was used for statistical analysis. **d** Representative images of Nox2 in brainstem of mouse brains treated with TM. Scale bar: 100 μm. Detail Scale bar: 20 μm. **e** The area of Nox2 in brainstem of mouse brains in (**d**) was quantified using IpWin32 software. *n* = 7 mice per group. Data are mean ± SEM, and a one-way ANOVA followed by Tukey’s multiple comparison test was used for statistical analysis. **f**, **h** Representative images of microglia (**f**) and astrocyte (**h**) in brainstem of mice with or without TM were detected by anti-Iba-1 and anti-GFAP antibodies, respectively. Scale bar: 100 μm. Detail scale bar: 20 μm. **g**, **i** Quantification of the area of Iba-1 positive microglia (**f**) and GFAP positive astrocyte (**h**) in brainstem of mice by IpWin32 software. *n* = 7 mice per group. Data are mean ± SEM, and a one-way ANOVA followed by Tukey’s multiple comparison test, or an unpaired *t* test with two-tailed was used for statistical analysis. **P < 0.01, ***P < 0.001, ****P < 0.0001, ns not significant.

We next detect the effects of TM on the levels of oxidative stress in the brains of A53T α-syn mice. The results showed that TM plasmids, but not vector control, significantly increased the levels of GSH and SOD, and reduced GSSG in A53T mice (Fig. 8a–c), while the level of oxidative stress in the shTM group was significantly up-regulated compared with that of the control group (Supplementary Fig. S13a–c). Similarly, the level of Nox2 in A53T mice were obviously decreased in TM overexpression group while it was increased in TM knockdown group (Fig. 8d, e and Supplementary Fig. S13d, e). Next, we detected neuroinflammation in A53T α-syn transgenic mice. IHC results showed that TM treatment significantly reduced microgliosis (Fig. 8f, g) and astrogliosis (Fig. 8h, i) in the brainstem of A53T α-syn mice compared with vector-treated mice, whereas shTM treatment significantly increased microgliosis and astrogliosis in A53T α-syn mice (Supplementary Fig. S13f–i). Consistently, these results were further confirmed in the striatum (Supplementary Fig. S14a–h). There was no significant effect observed in WT mice treated with TM.

## Discussion

There are 7 million people suffering from PD in the world, and the number of PD patients will double by 2040 [21]. Currently available treatments offer good control of motor symptoms but do not halt PD progression [22]. α-syn oligomers are considered a major risk factor for neuronal deterioration in PD. Discovering the factors associated with α-syn generation, aggregation, neurotoxicity, and the related underlying mechanism could improve our understanding of pathological processes of PD and help identify the targets of interest.

To protect the host from injury and promote healing, TM is given multiple functions associated with coagulation, inflammation, innate immunity, and cell proliferation [23]. The “traditional” role of TM is anticoagulation, while its many other novel functions were increasingly found. TM could activate PC, and the activated PC slowed the progression of ALS-like disease in mice by inhibiting SOD1 synthesis [9–11]. In this study, we found that the levels of TM were significantly decreased in the plasma or/and brains of PD patients and A53T mice. Consistently, our present results demonstrated that α-syn oligomers activated IL-1β signaling pathway, decreased the expression of KLF2, and ultimately reduced TM production, which was consistent with previous reports that identified KLF2 as a transcription factor for TM [24–26].

The increased expression of α-syn is one of characteristics in the brains of PD patients, but the factors influencing its expression remain largely unknown. α-syn level can be regulated by transcriptional and posttranscriptional mechanisms, which include transcription factors, microRNAs, splicing and posttranslational degradation. A signaling pathway involving Erk/PI3 was previously reported to induce α-syn transcription [5, 27]. Consistently, our present in vitro and in vivo results showed that decreased TM activated Erk1/2 signaling pathway, and increased α-syn expression, while overexpressed TM inhibited α-syn expression by decreasing p-c-Jun level through Erk1/2 signaling pathway. The decreased TM in PD patients or mouse models remarkably promotes α-syn expression, and then higher level of α-syn reduces TM generation, forming a vicious circle. Knockdown TM in A53T mice strengths this circle, inducing more severe neuropathology and motor deficits, whereas overexpressed TM breaks this circular pattern, reducing α-syn generation and ameliorating PD-like pathology and symptoms.

Oxidative stress is caused by an imbalance between the production and detoxification of ROS. In this study, α-syn increased the ROS production by inhibiting the anti-oxidative activities of SOD, exhibiting reduced GSH levels and increased GSSG levels in primary neurons, and this α-syn-induced oxidative stress was significantly inhibited by TM through restoring SOD activities, and increasing GSH levels. Consistent with these results in vitro, TM significantly enhanced the levels of SOD and GSH, and decreased Nox2 levels in A53T α-syn mice (Fig. 8). Intrinsic apoptotic (mitochondria-mediated) pathway activated by α-syn is a predominant mode of neuronal death in PD [28], which is mediated by the proteins that possess either proapoptotic such as Bax or anti-apoptotic such as Bcl-2 and the permeability transition pore [29]. Enhanced Bax levels, elevated activity and expression of caspase-3, and decreased Bcl-2 levels were found in substantia nigra pars compacta in postmortem of PD patients [30–32]. In this study, we found that TM reduced α-syn-medicated apoptosis through regulating p53 expression, the Bax/Bcl-2 ratio, and caspase-3 activity in neurons via PAR1-p53-Bax signaling pathway (Fig. 3), which is consistent with the previous report that TM dependent PC activation protects neuronal cells from cell death [11].

In summary, present investigation applying both specific overexpression and knockdown of TM in neurons demonstrates that TM decreases α-syn generation, neuronal apoptosis, gliosis and oxidative stress, and improves DA production, and the motor and memory deficits via multiple signal pathways in a transgenic mouse model of PD. Our in vitro and in vivo experimental results markedly confirm the beneficial effect of TM on PD pathology, and suggest that TM is a potential target for alleviating pathological features and treating PD.

## Materials and methods

### Animals

A53T α-syn mice (Strain#: 004479) were originally obtained from Jackson Laboratory, and kept in the animal facility of Tsinghua University. Then A53T α-syn male mice and their C57BL/6 male littermates were generated by breeding A53T α-syn male mice with wild-type female mice under the original C57BL/6× C3H background. 10-month-old male C57BL/6 or A53T heterozygous mice, weighing 32–38 g, mice were randomly assigned to different experimental group.

All mice were given food and water ad libitum and kept in a colony room at 22 ± 2 °C temperature and 45% ± 10% humidity under a 12 h:12 h light/dark cycle. Experiments involving mice and protocols were approved by the Institutional Animal Care and Use Committee of Tsinghua University.

### Subjects and sample collection

We collected plasma samples from PD patients (*n* = 30) with a median age of 67 years (range, 46–81 years), and 15 control subjects, with a median age of 64 years (range, 55–75 years). Patient’s information including age, sex and clinical diagnosis was shown in Supplementary Table S1. All the patients were diagnosed primary Parkinson’s Disease according to MDS Clinical Diagnostic Criteria for Parkinson’s Disease Exclusion criteria were as follows: atypical or secondary Parkinsonism; a confounding medical or psychiatric condition (s); any condition that precludes the ability to provide informed consent; and other neurologic diseases leading to motor deficits. Community volunteers with no neurological and/or psychiatric conditions were recruited as healthy controls. The level of TM in human plasma was detected by Human TM protein ELISA Kit (KA2250, Abnova) according to the manufacturer’s instructions. This study was approved by the Institutional Review Board of the Beijing Friendship Hospital, Capital Medical University. All subjects signed written informed consent before their enrolment in the study.

### Expression and purification of α-syn

α-syn was expressed and prepared according to the previous reports [33]. α-syn oligomers were prepared as described by Pieri et al. [34]. Briefly, freshly prepared α-syn was diluted to 1 mg/mL with PBS, then incubated at 37 °C under constant stirring at 200 rpm. The aggregation states of α-syn were determined by Thioflavin T fluorescence assay. For fibril preparation, α-syn monomers were solubilized in PBS at the concentration of 5 mg/mL and incubated at 37 °C shaking for 7 days [35]. Then, the fibrils were sonicated 10 min using a water bath sonicator.

### Production of recombinant lentiviral vectors

We used lentiviruses carrying TM shRNA (shTM) or TM plasmid (TM) to reduce or upregulate TM expression, respectively [36, 37]. The packaging constructs pSicor or pCDH was used for downregulation or overexpression, respectively, and the transfer vectors expressing the transgenes were PsPax2 and pMD.2 G. The 293 T cells were transfected with pSicor or PCDH, PsPax2 and pMD.2 G for 48 h to produce virus particles, and the supernatant was collected, filtered and centrifuged to obtain high titer stock solution. The precipitate was re-suspended in PBS and stored at −80 °C. The virus was tested for the absence of virus vectors capable of replication. The final virus concentration was calculated by qPCR assay.

### Primary neuronal cultures

Primary neuronal cell cultures were prepared from 14-to 15-day-old C57BL/6 embryonic mice. Cortex tissue was first isolated from fetal mouse brains, cut and treated with 0.25% trypsin-EDTA (containing 1 mg/ml DNase I, Thermo Fisher Scientific, #90083) for 15 min and then neutralized with DMEM (Gibco, #11965092) and 10% FBS (Sigma-Aldrich, #F8318), and the cell suspension was filtered through a 70 μm cell filter. After centrifugated at 1500×*g* for 10 min at 4 °C, cells were plated at a density of 150,000/well in 12-well petri dishes on poly-D-lysine coated coverslip and cultured in neuro-based medium containing B27 (Thermo Fisher Scientific, #17504044) and l-glutamine (Thermo Fisher Scientific, #35050061). The medium was changed every 2–3 days. For the α-syn degradation experiments, primary neurons were infected with TM or Vector for 48 h, and then treated with 100 μg/mL CHX and harvested at different time points.

### Cell culture

PC12 cells was kindly provided by Prof. Nai-hong Chen from Institute of Materia Medica, Chinese Academy of Medical Science & Peking Union Medical College. 293T cells were purchased from ATCC (CRL-3216). PC12 cells and 293T cells were cultured in DMEM medium (Gibco, #C11965500CP) supplemented with fetal bovine serum (FBS) (10%, Gibco, #10099141), and 0.5% penicillin and streptomycin in 5% CO_2_ at 37 °C and regularly monitored for mycoplasma infections.

### Plasmid, Small interfering (si) RNA and transfection

The TM gene was linked to the pmCherry-C1 plasmid to construct the overexpressed plasmid, and the plasmid without TM gene was used as a control. TM siRNA (5’-GGUGCGAAAUGUUCUGCAATT-3’) and NC were purchased from Shanghai GenePharma Co., Ltd. (Shanghai, China). PC12 cells were plated at 70% confluence and transfected with siRNA or TM plasmid using Lipofectamine 3000 (Thermo Fisher Scientific, #L3000015) according to the manufacturer’s protocol.

### Lentivirus infection

In order to explore the effect of TM on the expression of α-syn, and the underlying mechanism, we reduced or increased the expression of TM in primary cortical neurons (DIV7) by infected with sh-TM or sh-NC, LV-TM or LV-Vector, respectively. Neurons were analyzed 72 h after infection (DIV10). In addition, 48 h after infection, the cells were treated by honokiol HNK (MedChemExpress, #HY-N0003) for 12 h to activate p-Erk1/2.

### Protein extractions

Mice were deeply anesthetized with sodium pentobarbital, transcardially perfused with ice-cold PBS containing heparin (10 U/mL), and finally sacrificed. Brainstem and striatum were harvested in 1 mL of RIPA buffer solution containing 1 mM PMSF, and protease inhibitor cocktail set I. Brain tissues were broken at a frequency of 30 Hz in Tissue Lyser II (Qiagen) for 8 min, and the homogenate were centrifuged at 14,000 rpm, 4 °C for 30 min, and the supernatants were collected and termed as soluble fraction. The pellets were dissolved in 10% SDS, and the homogenates were centrifuged at 10,000 rpm for 30 min. The new supernatants were considered to be the insoluble fraction. The protein concentrations of soluble and insoluble fractions were determined using Pierce™ BCA Protein Assay Kit (Thermo Fisher Scientific, #23225) according to the manufacturer’s instructions.

### Western blot analysis

For western blotting assay, proteins from cells were lysed using Biyuntian Membrane protein Extraction Kit. Protein samples were electrophoresed on 12% SDS-PAGE gels and transferred to nitrocellulose membranes. After blocking with 5% skim milk at room temperature for 1 h, appropriate antibodies were added and incubated at 4 °C overnight. The next day, the membranes were washed three times with TBST for 10 min each time and incubated with related secondary antibodies for 1 h at room temperature. The membranes were then washed three more times and imaged in an Amersham imaging system (GE Healthcare, Piscataway, USA). Each target protein was subjected to 3–4 separate experiments under the same conditions and quantified using Image J software.

The following primary antibodies were used for western blotting assay in this study: anti-Thrombomodulin antibody (#ab230010, Abcam, 1:1000), anti-Tyrosine Hydroxylase antibody (#ab137869, Abcam, 1:1000), anti-Dopamine Transporter antibody (#ab184451, Abcam, 1:1000), anti-Alpha-synuclein antibody (#ab27766, Abcam, 1:1000), anti-phospho-Alpha-synuclein antibody (#ab51253, Abcam, 1:1000), anti-RAGE antibody (#AF5309, Affinity, 1:1000), anti-phospho-Erk1 (pT202/pY204) + Erk2 (pT185/pY187) antibody (#ab76299, Abcam, 1:1000), anti-Erk1/2 antibody (#ab184699, Abcam, 1:1000), anti-c-Jun antibody (#AF1612, Beyotime, 1:1000), anti-phospho-c-Jun (Ser73) antibody (#AF5782, Beyotime, 1:1000), anti-P65 antibody (#ab32536, Abcam, 1:1000), anti-APC antibody (#AF2113, Beyotime, 1:1000), anti-PAR-1 antibody (#bs-0828F, Bioss, 1:1000), anti-p38 antibody (#AM-065-1, Beyotime, 1:1000), anti-APC antibody (#AF2113, Beyotime, 1:1000), anti-phospho-P38 antibody (#AM-063, Beyotime, 1:1000), anti-p53 antibody (#ab131442, Abcam, 1:1000), anti-caspase9 antibody (#HY-P80050, MCE, 1:1000), anti-Cleaved caspase9 antibody (#HY-P80964, MCE, 1:1000), anti-caspase3 antibody (#AF1213, Beyotime, 1:1000), anti-Cleaved caspase3 antibody (#AC033, Beyotime, 1:1000), anti-Bax antibody (#ab182734, Abcam, 1:1000), anti-Bcl-2 antibody (#ab182858, Abcam, 1:1000), anti-β actin antibody (#TA-09, ZSGB-BIO, 1:1000).

### Quantitative real-time PCR analysis

RNA was extracted from cell lysates or brain homogenates using the TRIzol according to the manufacturer’s instructions. cDNA was prepared from total RNA using the EasyQuick RT MasterMix (#CW2019M, Cwbio, China). Relative gene amplification of the cDNA was assayed using PE ABI PRISM® 7700 Sequence Detection System (PErkin Elmer, Wellesley, MA, USA) and SYBR Green method following manufacturer’s instructions. Finally, analysis was performed using 2^-ΔΔct^.

The used primers in this study are described as follows:

TM:

5’-AGGGAAGACACCAAGGAAGAGGAG-3’

5’-GGACAGGCTGGCAATGGAGATG-3’

APC:

5’-TGTGAGTGAATGATGTTGTGGAGTG-3’

5’-TGCGGCAGTCTGGATGTCTTC-3’

PAR-1:

5’-GTCAATGGGTTACACACGGGAAGAG-3’

5’-AGCCAAGGAGCAGATAGGTAGCC-3’

Erk1/2:

5’-GCCTTCCAACCTCCTGCTGAAC-3’

5’-CGTACTCTGTCAAGAACCCTGTGTG-3’

c-Jun:

5’-CTTCTACGACGATGCCCTCAACG-3’

5’-GCCAGGTTCAAGGTCATGCTCTG-3’

α-syn:

5’-TGGCTTTGTCAAGAAGGACCAGATG-3’

5’-CCACAGGCATGTCTTCCAGGATTC-3’

RAGE:

5’-GAGTCTGGGCTGGGTACGCTAG-3’

5’-TTCCTCCTCATCCTGGCTTTC-3’

p38:

5’-CTGGCTCGGCACACTGATGATG-3’

5’-GCCCACGGACCAAATATCCACTG-3’

p53:

5’-ACCGCCGACCTATCCTTACCATC-3’

5’-GGCACAAACACGAACCTCAAGC-3’

Bax:

5’-GCTACAGGGTTTCATCCAGGATCG-3’

5’-TGCTGTCCAGTTCATCTCCAATTCG-3’

Bcl-2:

5’-GGTGGACAACATTGCCCTCTG-3’

5’-AAAGACAGCCAGGAGAAGTCAAAC-3’

β-actin:

5’-AAGAGGGATGCTGCCCTTAC-3’

5’-TACGGCCAAATCCGTTCACA-3’

### ELISA

The levels of α-syn, mouse TM, mouse Dopamine, mouse homovanillic acid (HVA) and mouse 3,4-dihydroxyphenyl acetic acid (DOPAC) in the mouse plasma, cell lysis supernatant or brain homogenate were detected following the manufacturer’s instructions. The ELISA kits used in this article are as follows: Human Alpha Synuclein ELISA Kit (EK3001, SAB Signal way Antibody), Mouse thrombomodulin ELISA Kit (M150964, Mreda), Mouse Dopamine ELISA Kit (EK10926, SAB), Mouse homovanillic acid (HVA) ELISA Kit (TQ9910, Tian Qishun), Mouse 3,4-dihydroxyphenyl acetic acid (DOPAC) ELISA Kit (DG91359Q, Dogesce).

### Cell toxicity assessment

MTT assay was used to detect the effect of TM on α-syn neurotoxicity. In brief, 10,000 primary neurons per well were seeded in 96-well plates and transfected with shTM, TM or control for 72 h, and then 3 μM α-syn or the same volume of incubation buffer was added to the cells for 72 h. Cell viability was determined by adding 25 μL of 5 mg/mL MTT to each well. After 3 h of incubation at 37 °C, the supernatants were replaced with 150 μL aliquot of DMSO in the dark. The absorbance at 570/630 nm was measured by using a SpectraMax M5 microplate reader (Molecular Devices, Sunnyvale, CA). Data were obtained from three independent experiments.

### Measurement of reactive oxygen species (ROS), superoxide dismutase (SOD) and GSH

The activity of ROS and SOD in primary neurons treated with shTM, TM or control was determined by a ROS assay kit (Beyotime Biotechnology, S0033M) or SOD assay kit (Beyotime biotechnology, S0109) according to the manufacturer’s protocol. Briefly, the cells were treated, and cell lysates were prepared as previously described [38]. The mixtures of the samples, DCFH-DA, nitroblue tetrazolium, and enzyme working solutions were incubated at 37 °C for 20 min, and their absorbance was assayed at 560 nm using the SpectraMax M5 microplate reader (Molecular Devices, Sunnyvale, CA).

GSH and GSSG levels in primary neurons treated with shTM, TM or Control were measured using a commercial kit (Beyotime, S0053). Total GSH was measured by a 5, 5-dithiodibis (2-nitrobenzoate) acid (DTNB) -GSSG reductase cycle. GSSG was obtained by measuring the absorbance of 5-thio2-nitrobenzoic acid generated by the reaction of reduced GSH with DTNB according to the manufacturer’s protocol. The reduced GSH was obtained by subtracting GSSG from total GSH. Absorbance was measured at 412 nm using the SpectraMax M5 microplate reader. The levels of SOD, GSH and GSSG in brain lysates of mice were also detected as described above.

### Animal and drug administration

All animal experiments were performed in accordance with the China Public Health Service Guide for the Care and Use of Laboratory Animals. Experiments involving mice and protocols were approved by the Institutional Animal Care and Use Committee of Tsinghua University (AP#15-LRT1).

Ten-month-old A53T heterozygous mice were injected with 5 µg of α-syn fiber into the bicep femoralis muscle bilaterally according to previous report [39]. Following fiber injections, mice in each group (*n* = 8) were deeply anesthetized with a mixture of ketamine (100 mg/kg) and xylazine (10 mg/kg), and bilaterally injected 1 × 10^10^ vg of AAV-TM, AAV-Vector, AAV-shTM, AAV-NC into the substantia nigra at 60 days post fiber injection. We used the following coordinates: anteroposteric (AP) = 3.3 mm, mediolateral (mL) =±1.5 mm, dorsoventral (DV) = −4.5 mm. The serotype of AAV was 2/8. The surgical site was cleaned with sterile saline and the incision was closed. After surgery, the animals are monitored and post-operative care is provided. At 30 days after stereotactic injection, mice were trained and tested for behavioral and cognitive abilities, the experimenters were double-blind in their group classification, and finally sacrificed for biochemical and histological analyses.

### Body suspension test

Muscle strength of mice was tested by body suspension test as previously described [40]. The animals were suspended by their forelimbs to a metal bar with a diameter of 2 mm and the time was measured until the animals lost the bar and dropped down. Mice were tested three times.

### Open field test

The open field test was performed according to a previously described method with slight modifications. Mice were placed individually in the center of the chamber (27 × 27 × 20.3 cm^3^) equipped with a camera. After a 1 min adaptation period, their behavior was recorded for 10 min. The distance in center travelled, total time in center were quantitatively analyzed. Chambers were cleaned with 70% ethanol between trials.

### Pole test

Mice were initially habituated and trained 1 d prior to testing. Then they were placed on the top of a rough-surfaced wooden pole (50 cm in length and 1 cm in diameter) and allowed to descend to the base of the pole. During the test, mice were placed with their heads oriented toward the top of the pole. The time required by the mouse to descend the entire length of the pole was measured. The best performance of each mouse over five consecutive trials was recorded.

### Rotarod test

Motor coordination of mice was assessed with a rotarod apparatus (TSE Systems). Daily sessions included a 5-min training trial at 4 rpm. After 1 h, mice were tested over three consecutive accelerating trials with the speed changing from 0 rpm to 40 rpm over 300 s. The inter-trial interval was 30 min. The latency to fall from the rod was recorded. Mice remaining on the rod for more than 300 s were removed, and their time was scored as 300 s. Mice were tested over three consecutive days.

### Y-maze test

The Y-maze test was conducted as previously described in detail. In brief, Y-maze testing consisted of two trials separated by an interval of 1 h. The first trial had a 10 min duration, and the mouse was allowed to explore only two arms (the start and familiar arms) of the maze with the third arm (novel arm) was blocked. In the second trial, the mice were put back in the starting arm with free access to all three arms for 5 min. By using a ceiling-mounted charge-coupled device camera, all trials were recorded on a videocassette recorder, and the number of entries and time spent in each arm in the video recordings were analyzed.

### Immunohistochemistry (IHC) and immunocytochemistry (ICC)

Mice were deeply anesthetized and immediately perfused with frozen PBS containing heparin (10 U/mL) before being sacrificed. The mouse brain was immediately removed and segmented along the sagittal plane. The right cerebral hemispheres were fixed in 4% paraformaldehyde at 4 °C overnight, and 5 μm sagittal sections were obtained using a Lecia CM1850 microtome. Prior to staining, sections were incubated with sodium citrate buffer (10 mM sodium citrate, pH 6.0) for 20 min at 95 °C to recover antigen. Next, sections were permeated and blocked with PBST containing 10% donkey serum and 0.3%Triton X-100 for 1 h at room temperature, then incubated with primary antibodies and followed by the corresponding fluorescently conjugated secondary antibodies and imaged on a Leica TCS SP8 confocal microscope. For 3’ diaminobenzidine (DAB) immunostaining, sections were incubated with primary antibodies, followed by corresponding HRP-labeled secondary antibodies, and DAB was visualized with an Olympus IX73 inverted microscope with a DP80 camera. All images were analyzed by Image J Software.

To detect neuronal damage and apoptosis, Nissl staining was carried out according to standard protocols (#G1430, Solarbio). Briefly, sections were incubated in Nissl solution, decolored in an alcohol series (70%, 95% and 100%; 20 s each), and finally immersed in xylene (three times for 5 min each). Moreover, a fluorescent terminal deoxynucleotidyl transferase nick-end labelling kit was used for TUNEL staining (Cat#C1088, Beyotime Biotechnology, Beijing, China). As a positive control, sections were incubated with recombinant DNase I.

For ICC assay, cells were fixed in 4% paraformaldehyde (PFA) for 20 min at room temperature, permeated with 0.3%Triton X-100 for 25 min, and blocked with blocking buffer (10% donkey serum in PBS) for 30 min. Cells were then cultured with primary antibodies for 1 h at room temperature. Then cells were washed three times with PBST, and incubated with the corresponding Alexa-conjugated secondary antibodies for 45 min at room temperature. Subsequently, the cells were incubated with Hoechst (Cat#94403-1 ML, Sigma-Aldrich, 1:1000) in dark places for 15 min, and fading-resistant mount media (Cat#P1026, Beyotime Biotechnology, Beyontime Biotechnology, Beijing, China) on a cover glass. Fluorescence signals were captured on a laser scanning confocal microscope (Leica TCS SP8, Germany).

The following primary antibodies were used for IHC and ICC assay in this study: anti-MAP2 antibody (#ab254143, Abcam, 1:200), anti-Iba-1 antibody (#ab283319, Abcam, 1:200), anti-GFAP antibody (#ab279289, Abcam, 1:200), anti-mCherry antibody (#ab125096, Abcam, 1:200), anti-Tyrosine Hydroxylase antibody (#ab137869, Abcam, 1:200), anti-Thrombomodulin antibody (#ab230010, Abcam, 1:200), anti-α-synuclein antibody (#ab51253, Abcam, 1:200), anti-Tyrosine Hydroxylase (#ab137869, Abcam, 1:200), anti- Nox2 antibody (#ab80897, Abcam, 1:200).

### Statistical analyses

All quantitative analyses were performed under blinded conditions. Detailed *n* values for each panel in the figures were stated in the corresponding legends. Sample sizes were based in standard protocols in the field and no sample or animal was excluded. All statistical analyses were performed by GraphPad Prism 8.0 Software. Student’s *t* test was used to compare data between two groups, and one-way analysis of variance (ANOVA) or two-way ANOVA followed by Bonferroni’s multiple comparison test was used to compare data between three or more groups. Results were expressed as group mean ± SEM, and *P* < 0.05 was considered statistically significant.

### Supplementary information

Supplemental Material

Original Data

## Data Availability

All data generated in this study is available in the main text or the supplementary Materials. Any additional information required to reanalyze the data reported in this paper is available from the lead contact upon request.

## References

[1] Silva RC, Domingues HS, Salgado AJ, Teixeira FG (2022). From regenerative strategies to pharmacological approaches: can we fine-tune treatment for Parkinson’s disease?. Neural Regen Res.

[2] Tansey MG, Wallings RL, Houser MC, Herrick MK, Keating CE, Joers V (2022). Inflammation and immune dysfunction in Parkinson disease. Nat Rev Immunol.

[3] Spillantini MG, Schmidt ML, Lee VM, Trojanowski JQ, Jakes R, Goedert M (1997). Alpha-synuclein in Lewy bodies. Nature.

[4] Oliveira LM, Falomir-Lockhart LJ, Botelho MG, Lin KH, Wales P, Koch JC (2015). Elevated alpha-synuclein caused by SNCA gene triplication impairs neuronal differentiation and maturation in Parkinson’s patient-derived induced pluripotent stem cells. Cell Death Dis.

[5] Lee Clough R, Stefanis L (2007). A novel pathway for transcriptional regulation of α‐synuclein. FASEB J.

[6] Erekat NS. Apoptosis and its role in Parkinson’s disease. In: Stoker TB, Greenland JC, editors. Parkinson’s disease: pathogenesis and clinical aspects. Codon Publications; 2018.

[7] Lee HJ, Suk JE, Patrick C, Bae EJ, Cho JH, Rho S (2010). Direct transfer of alpha-synuclein from neuron to astroglia causes inflammatory responses in synucleinopathies. J Biol Chem.

[8] Kim C, Ho D-H, Suk J-E, You S, Michael S, Kang J (2013). Neuron-released oligomeric α-synuclein is an endogenous agonist of TLR2 for paracrine activation of microglia. Nat Commun.

[9] Cheng T, Liu D, Griffin JH, Fernández JA, Castellino F, Rosen ED (2003). Activated protein C blocks p53-mediated apoptosis in ischemic human brain endothelium and is neuroprotective. Nat Med.

[10] Zhong Z, Ilieva H, Hallagan L, Bell R, Singh I, Paquette N (2009). Activated protein C therapy slows ALS-like disease in mice by transcriptionally inhibiting SOD1 in motor neurons and microglia cells. J Clin Investig.

[11] Guo H, Liu D, Gelbard H, Cheng T, Insalaco R, Fernández JA (2004). Activated protein C prevents neuronal apoptosis via protease activated receptors 1 and 3. Neuron.

[12] Shahzad K, Kohli S, Isermann B (2019). Cell biology of activated protein C. Curr Opin Hematol.

[13] Wolter J, Schild L, Bock F, Hellwig A, Gadi I, Al‐Dabet M (2016). Thrombomodulin‐dependent protein C activation is required for mitochondrial function and myelination in the central nervous system. J Thromb Haemost.

[14] Saito H, Maruyama I, Shimazaki S, Yamamoto Y, Aikawa N, Ohno R (2007). Efficacy and safety of recombinant human soluble thrombomodulin (ART‐123) in disseminated intravascular coagulation: results of a phase III, randomized, double‐blind clinical trial. J Thromb Haemost.

[15] Vincent J-L, Ramesh MK, Ernest D, LaRosa SP, Pachl J, Aikawa N (2013). A randomized, double-blind, placebo-controlled, Phase 2b study to evaluate the safety and efficacy of recombinant human soluble thrombomodulin, ART-123, in patients with sepsis and suspected disseminated intravascular coagulation. Crit Care Med.

[16] Monje P, Hernandez-Losa J, Lyons RJ, Castellone MD, Gutkind JS (2005). Regulation of the transcriptional activity of c-Fos by ERK. A novel role for the prolyl isomerase PIN1. J Biol Chem.

[17] Qu D, Ling Z, Tan X, Chen Y, Huang Q, Li M (2019). High mobility group protein B1 (HMGB1) interacts with receptor for advanced glycation end products (RAGE) to promote airway smooth muscle cell proliferation through ERK and NF-kappaB pathways. Int J Clin Exp Pathol.

[18] Chen J, Ren Y, Gui C, Zhao M, Wu X, Mao K (2018). Phosphorylation of Parkin at serine 131 by p38 MAPK promotes mitochondrial dysfunction and neuronal death in mutant A53T alpha-synuclein model of Parkinson’s disease. Cell Death Dis.

[19] Guo JD, Zhao X, Li Y, Li GR, Liu XL (2018). Damage to dopaminergic neurons by oxidative stress in Parkinson’s disease (review). Int J Mol Med.

[20] Kagawa Y, Matsumoto S, Kamioka Y, Mimori K, Naito Y, Ishii T (2013). Cell cycle-dependent Rho GTPase activity dynamically regulates cancer cell motility and invasion in vivo. PLoS ONE.

[21] Dorsey E, Bloem B (2018). The Parkinson pandemic—a call to action. JAMA Neurol.

[22] Bloem BR, Okun MS, Klein C (2021). Parkinson’s disease. Lancet.

[23] Loghmani H, Conway EM (2018). Exploring traditional and nontraditional roles for thrombomodulin. Blood J Am Soc Hematol.

[24] Dekker RJ, Boon RA, Rondaij MG, Kragt A, Volger OL, Elderkamp YW (2006). KLF2 provokes a gene expression pattern that establishes functional quiescent differentiation of the endothelium. Blood.

[25] Lin Z, Kumar A, SenBanerjee S, Staniszewski K, Parmar K, Vaughan DE (2005). Kruppel-like factor 2 (KLF2) regulates endothelial thrombotic function. Circ Res.

[26] SenBanerjee S, Lin Z, Atkins GB, Greif DM, Rao RM, Kumar A (2004). KLF2 Is a novel transcriptional regulator of endothelial proinflammatory activation. J Exp Med.

[27] Clough RL, Dermentzaki G, Stefanis L (2009). Functional dissection of the α‐synuclein promoter: transcriptional regulation by ZSCAN21 and ZNF219. J Neurochem.

[28] Anglade P, Vyas S, Javoy-Agid F, Herrero M, Michel P, Marquez J (1997). Apoptosis and autophagy in nigral neurons of patients with Parkinson’s disease. Histol Histopathol.

[29] Marzo I, Brenner C, Zamzami N, Susin SA, Beutner G, Brdiczka D (1998). The permeability transition pore complex: a target for apoptosis regulation by caspases and Bcl-2–related proteins. J Exp Med.

[30] Mogi M, Togari A, Kondo T, Mizuno Y, Komure O, Kuno S (2000). Caspase activities and tumor necrosis factor receptor R1 (p55) level are elevated in the substantia nigra from Parkinsonian brain. J Neural Trans.

[31] Viswanath V, Wu Y, Boonplueang R, Chen S, Stevenson FF, Yantiri F (2001). Caspase-9 activation results in downstream caspase-8 activation and bid cleavage in 1-methyl-4-phenyl-1, 2, 3, 6-tetrahydropyridine-induced Parkinson’s disease. J Neurosci.

[32] Hartmann A, Hunot S, Michel PP, Muriel M-P, Vyas S, Faucheux BA (2000). Caspase-3: a vulnerability factor and final effector in apoptotic death of dopaminergic neurons in Parkinson’s disease. Proc Natl Acad Sci USA.

[33] Hoyer W, Antony T, Cherny D, Heim G, Jovin TM, Subramaniam V (2002). Dependence of α-synuclein aggregate morphology on solution conditions. J Mol Biol.

[34] Pieri L, Madiona K, Melki R (2016). Structural and functional properties of prefibrillar alpha-synuclein oligomers. Sci Rep.

[35] Polinski NK, Volpicelli-Daley LA, Sortwell CE, Luk KC, Cremades N, Gottler LM (2018). Best practices for generating and using alpha-synuclein pre-formed fibrils to model Parkinson’s disease in rodents. J Parkinsons Dis.

[36] Hottinger AF, Azzouz M, Deglon N, Aebischer P, Zurn AD (2000). Complete and long-term rescue of lesioned adult motoneurons by lentiviral-mediated expression of glial cell line-derived neurotrophic factor in the facial nucleus. J Neurosci.

[37] Huang Y-R, Xie X-X, Yang J, Sun X-Y, Niu X-Y, Yang C-G (2023). ArhGAP11A mediates amyloid-β generation and neuropathology in an Alzheimer’s disease-like mouse model. Cell Rep.

[38] Zhou WW, Lu S, Su YJ, Xue D, Yu XL, Wang SW (2014). Decreasing oxidative stress and neuroinflammation with a multifunctional peptide rescues memory deficits in mice with Alzheimer disease. Free Radic Biol Med.

[39] Sacino AN, Brooks M, Thomas MA, McKinney AB, Lee S, Regenhardt RW (2014). Intramuscular injection of alpha-synuclein induces CNS alpha-synuclein pathology and a rapid-onset motor phenotype in transgenic mice. Proc Natl Acad Sci USA.

[40] Metz GA, Schwab ME (2004). Behavioral characterization in a comprehensive mouse test battery reveals motor and sensory impairments in growth-associated protein-43 null mutant mice. Neuroscience.

