# Supplementary material for: Thrombomodulin reduces α-synuclein generation and ameliorates neuropathology in a mouse model of Parkinson’s disease
Source: Cell Death Discov. 2024 Apr 8;10:167. doi: 10.1038/s41420-024-01939-y (PMC11002034; doi:10.1038/s41420-024-01939-y)
Supplement: Supplementary file 1 — Supplemental Material [file 41420_2024_1939_MOESM1_ESM.docx]

**Thrombomodulin reduces α-synuclein generation and ameliorates neuropathology in a mouse model of Parkinson’s disease**

Xiao-yun Niu^1,2^†, Xi-xiu Xie^2^†, Hou-zhen Tuo^3,^†, Cui-ping Lv^2^, Ya-ru Huang^2^, Jie Zhu^2^, Shi-yu Liang^2^, Xiao-yu Du^2^, Cheng-gang Yang^4^, Sheng-jie Hou^2^, Xiao-ying Sun^2^, Ling-jie Li^2^, Fang Cui^2^, Qi-xin Huang^2^, Ying-bo Jia^2^, Yu-jiong Wang^1^*, Rui-tian Liu^2^*

^1^ College of Life Science, Ningxia University, Yinchuan 750021, Ningxia, China

^2^ National Key Laboratory of Biochemical Engineering, Institute of Process Engineering, Chinese Academy of Sciences, Beijing 100190, China

^3^ Department of Neurology, Beijing Friendship Hospital, Capital Medical University, Beijing, 100050, China

^4^ Department of BigData, Beijing Medintell Bioinformatic Technology Co., LTD, Beijing 100081, China

†These authors contributed equally to this work.

*Corresponding author

Yu-jiong Wang

College of Life Science, Ningxia University.

Helan Road, Yinchuan 750021, Ningxia, China

Tel.: +86 951 2062051; Fax: +86 951 2062051

E-mail: wyj@nxu.edu.cn

Rui-tian Liu

National Key Laboratory of Biochemical Engineering,

Institute of Process Engineering, Chinese Academy of Sciences.

Haidian District, Beijing 100190, China

Tel.: +86 10 82545017; Fax: +86 10 82544904

E-mail: [rtliu@ipe.ac.cn](mailto:rtliu@ipe.ac.cn)

**
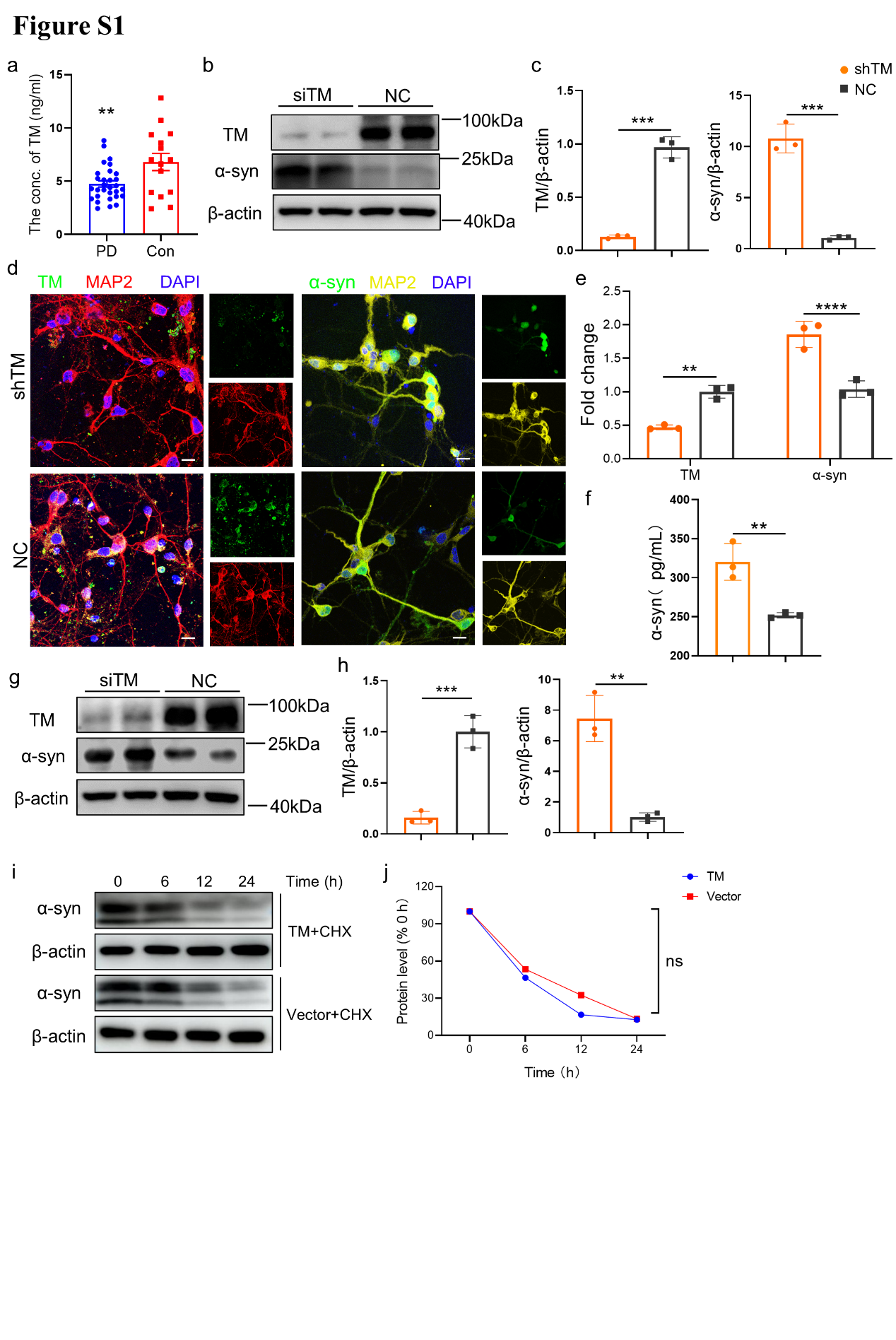
**

**Figure S1. TM downregulation increases α-syn levels in neurons. (a)** The levels of TM in the plasma of PD patients. The concentrations of TM in the plasma of PD patients (n=30) and healthy aged people (Con, n=15) were detected by ELISA kit. Data are mean ± SEM, and an unpaired t-test with two-tailed was used for statistical analysis. **(b)** Representative Western blotting of TM and α-syn in the PC12 cells transfected with TM small interfering (si) RNA (siTM). **(c)** Densitometry analysis of TM and α-syn in (b). n = 3 represents three independent experiments. Data are mean ± SEM, and an unpaired t-test with two-tailed was used for statistical analysis. **(d)** Representative confocal images of TM (green) and α-syn (green) in the neurons (DIV10, left panel: red; right panel: yellow) transfected with TM small hairpin RNA (shTM) or NC. Scale bar represents 10 μm. **(e)** The fluorescent area of TM and α-syn in (d) was quantified by IpWin32 software. n = 3 represents three independent experiments. Data are mean ± SEM, and an unpaired t-test with two-tailed was used for statistical analysis. **(f)** The levels of α-syn in primary neurons (DIV10) treated with shTM or NC was determined by ELISA. n = 3 represents three independent experiments. Data are mean ± SEM, and an unpaired t-test with two-tailed was used for statistical analysis. **(g)** Representative Western blotting of TM and α-syn in the primary neurons treated with shTM plasmid (shTM). **(h)** Densitometry analysis of TM and α-syn in (g). n = 3 represents three independent experiments. Data are mean ± SEM, and an unpaired t-test with two-tailed was used for statistical analysis. Data are mean ± SEM, and a one-way ANOVA followed by Tukey’s multiple comparison test. **(i)** The degradation of α-syn was assessed by half-life measurements in TM-infected primary neurons treated with CHX. **(j)** Relative levels of α-syn in (i) at different time points were quantified using Image J software. n = 3 is representative of three independent experiments. Data are mean ± SEM, and a two-way ANOVA followed by Tukey’s multiple comparison test was used for statistical analysis. * *P* < 0.05, ***P* < 0.01, ****P* < 0.001, *****P* < 0.0001, ns, not significant.


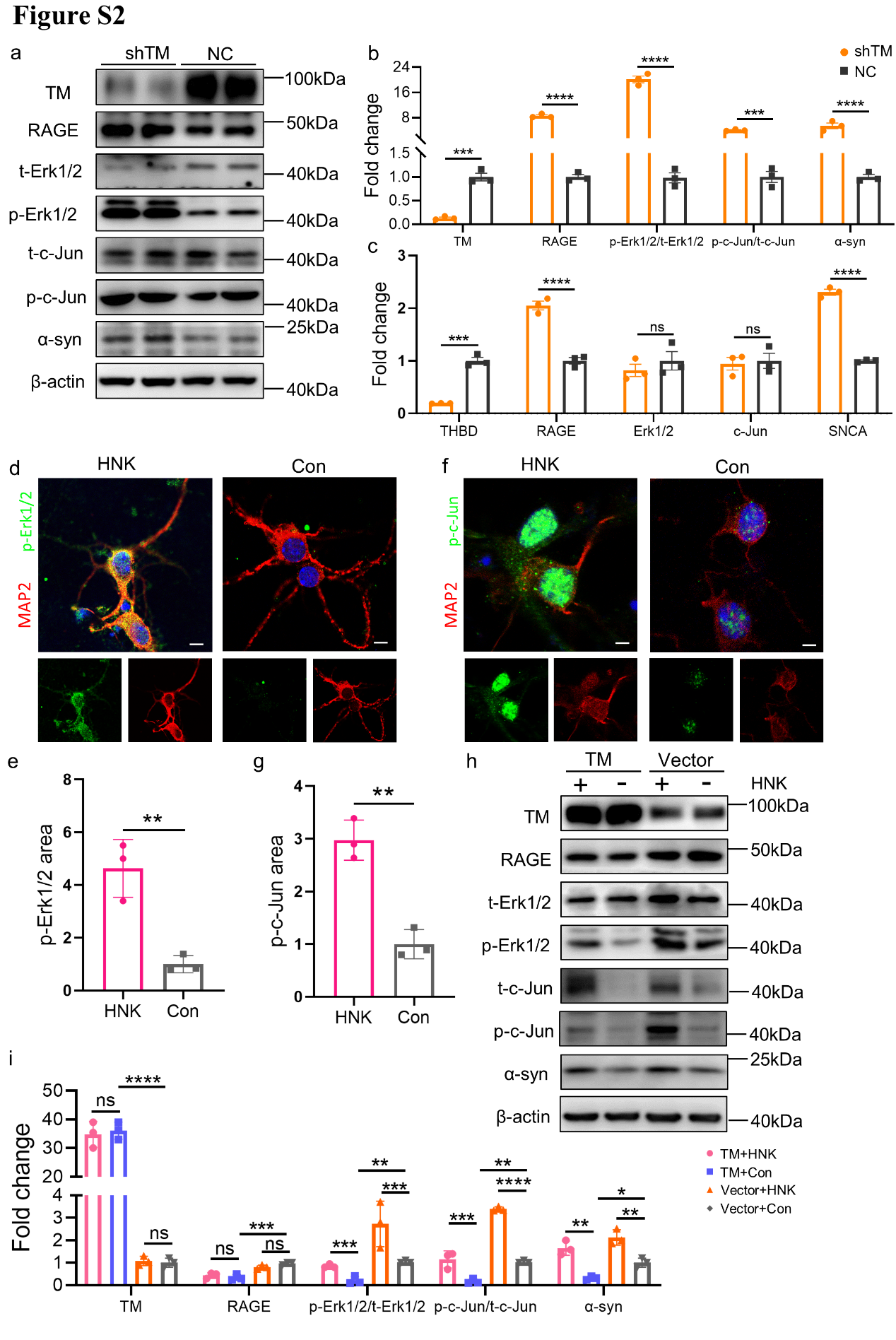


**Figure S2. p-Erk activation prevents the effect of TM on the expression of α-syn levels in neurons. (a)** The levels of TM, RAGE, t-Erk, p-Erk1/2, t-c-Jun, p-c-Jun and α-syn in primary neurons with shTM or NC were analyzed by Western blotting. β-actin was used as a control. **(b)** Relative levels of TM, RAGE, t-Erk1/2, p-Erk1/2, t-c-Jun, p-c-Jun and α-syn in (a) were quantified using Image J software. n = 3 represents three independent experiments. Data are mean ± SEM, and a one-way ANOVA followed by Tukey’s multiple comparison test. **(c)** The mRNA levels of TM, RAGE, Erk1/2, c-Jun and α-syn in primary neurons with shTM or NC were detected by qPCR. n = 3 represents three independent experiments. Data are mean ± SEM, and a one-way ANOVA followed by Tukey’s multiple comparison test was used for statistical analysis. **(d, f)** Representative confocal images of p-Erk1/2 (green) and p-c-Jun (green) in the primary cortical neurons (red) (DIV10) treated with HNK at 30 μM. Scale bar represents 5 μm. **(e, g)** The fluorescent area of p-Erk1/2 in (d) and p-c-Jun in (f) were quantified by IpWin32 software. n = 3 represents three independent experiments. Data are mean ± SEM, and an unpaired t-test with two-tailed was used for statistical analysis. **(h)** The levels of TM, RAGE, t-Erk1/2, p-Erk1/2, t-c-Jun, p-c-Jun and α-syn in lysates of primary cortical neurons (DIV10) infected with TM overexpression or control and treated with HNK were analyzed by Western blotting. **(i)** Relative levels of TM, RAGE, t-Erk1/2, p-Erk1/2, t-c-Jun, p-c-Jun and α-syn in (h) were quantified using Image J software. n = 3 represents three independent experiments. Data are mean ± SEM, and a one-way ANOVA followed by Tukey’s multiple comparison test was used for statistical analysis. **P* < 0.05, ***P* < 0.01, ****P* < 0.001, *****P* < 0.0001, ns, not significant.


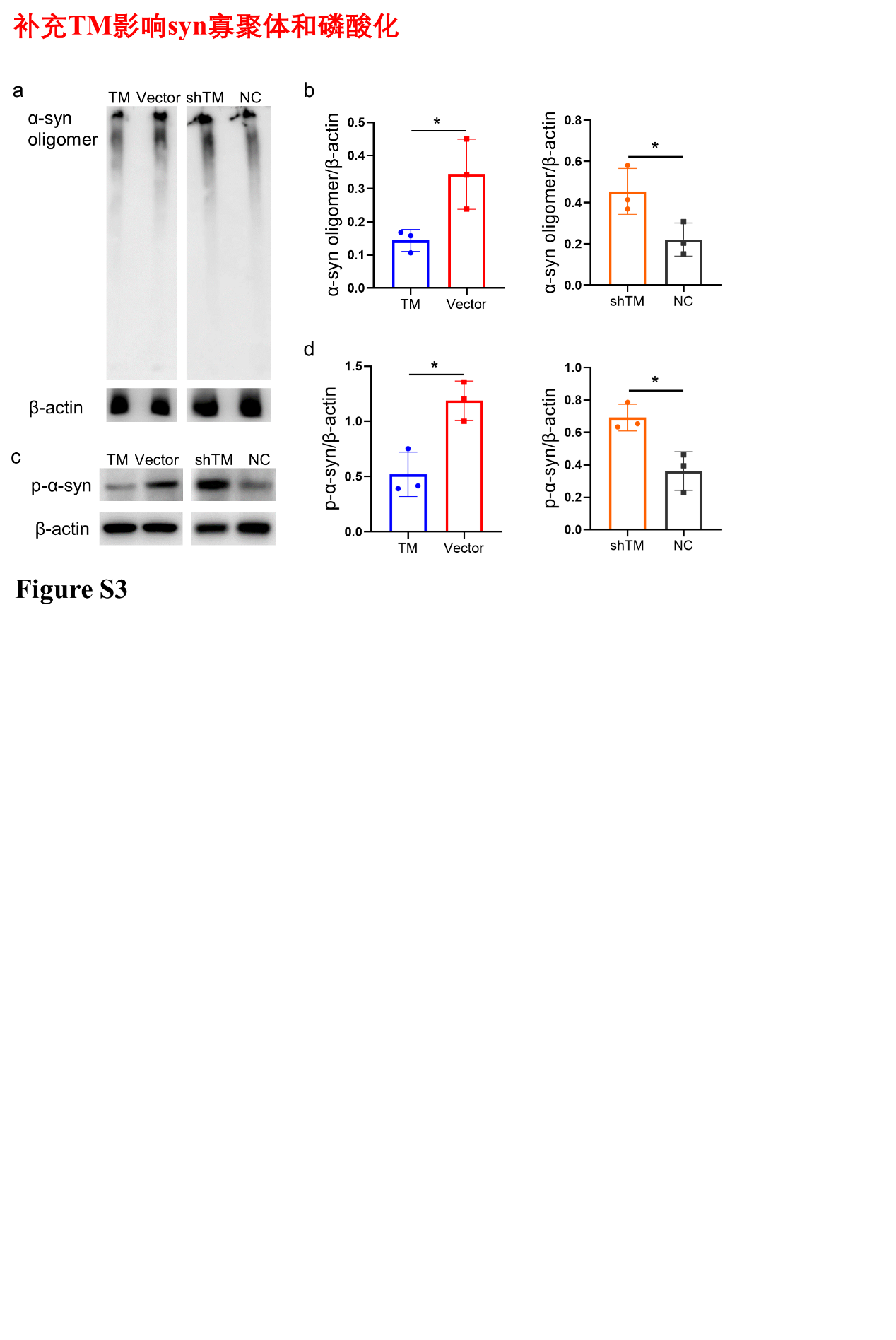


**Figure S3. TM affects the levels of** **α-syn aggregation and phosphorylation.** **(a, c)** The levels of α-syn aggregation (a) and phosphorylation (c) in primary cortical neurons (DIV10) treated with TM plasmid or shTM plasmid were analyzed by Western blotting. **(b, d)** Relative levels of α-syn oligomers in (a) and phosphorylated α-syn in (c) were quantified using Image J software. n = 3 represents three independent experiments. Data are mean ± SEM, and an unpaired t-test with two-tailed was used for statistical analysis. **P* < 0.05.


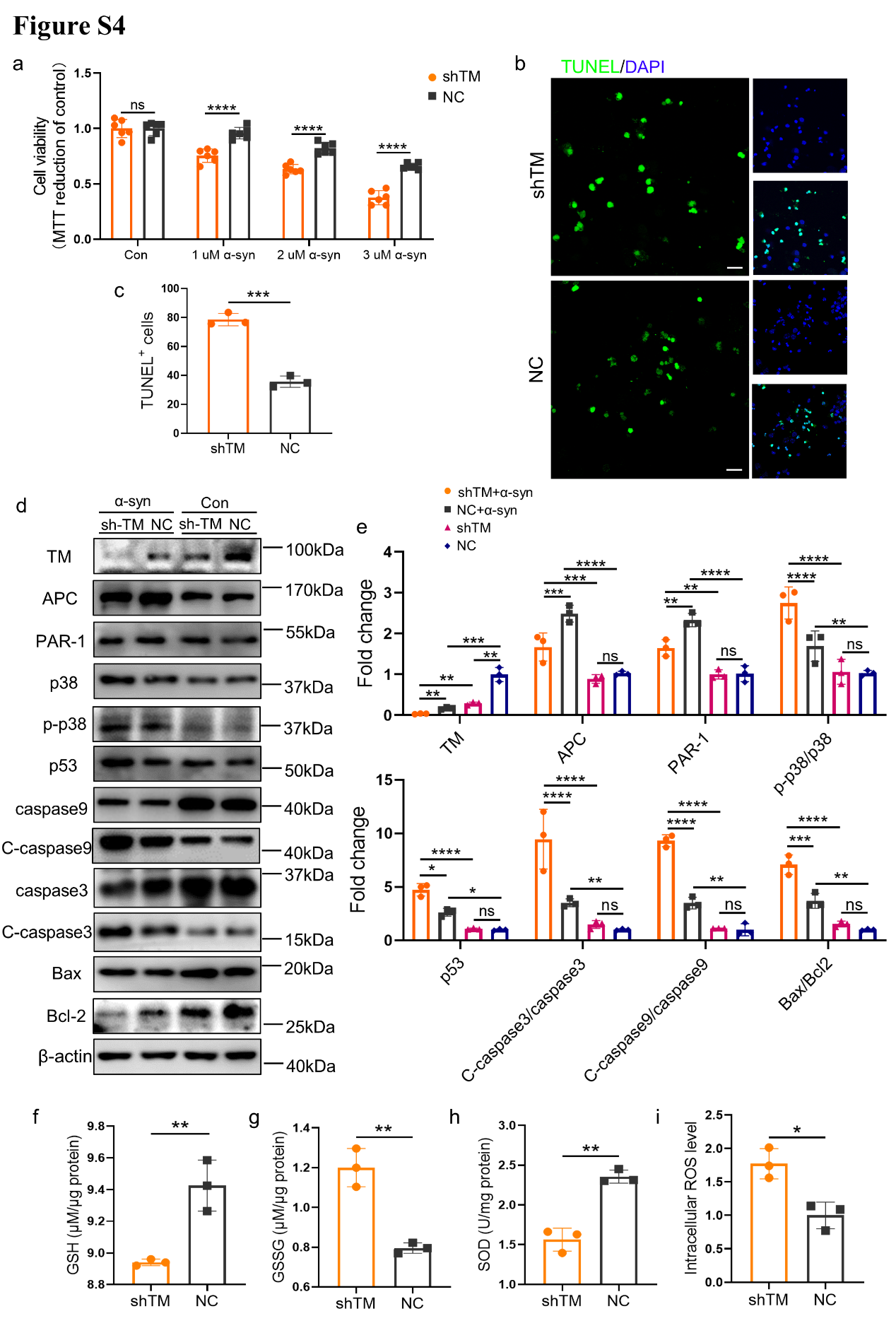


**Figure S4. TM downregulation enhances the levels of cell apoptosis and oxidative stress. (a)** The effect of shTM on the viability of primary neurons. Primary neurons (DIV8) infected with shTM or NC were treated with different concentration of α-syn oligomers for 72 h, and then cells was detected by MTT assay. n = 6 represents technical replicates. Experiment was repeated at least 3 times. Data are mean ± SEM, and an unpaired t-test with two-tailed was used for statistical analysis. **(b)** Primary neurons (DIV8) were treated with 2 μM α-syn oligomers for 72 h after infected with shTM or NC, then the cells were detected by TUNEL method. Scale bar represents 25 μm. **(c)** The fluorescent area of TUNEL^+^ cells in (b) were quantified by IpWin32 software. n = 3 represents three independent experiments. Data are mean ± SEM, and an unpaired t-test with two-tailed was used for statistical analysis. **(d)** The levels of TM, APC, RAR-1, p38, p-p38, p53, caspase9, Cleved-caspase9 (C-caspase9), caspase3, Cleved-caspase3 (C-caspase3), Bax and Bcl-2 in primary neurons infected with shTM after treated with 2 μM α-syn oligomers. β-actin was used as a control. **(e)** Relative levels of TM, APC, PAR-1, p38, p-p38, p53, caspase9, C-caspase9, caspase3, C-caspase3, Bax and Bcl-2 in (d) were quantified using Image J software, respectively. n = 3 represents three independent experiments. Data are mean ± SEM, and a one-way ANOVA followed by Tukey’s multiple comparison test. **(f-i)** The effect of shTM or NC on the levels of oxidative stress (GSH, GSSG, SOD and ROS). Primary neurons infected with shTM or NC was incubated with 2 μM α-syn oligomers for 48 h. Then the levels of oxidative stress were detected by ELISA kit. n = 3 represents three independent experiments. Data are mean ± SEM, and an unpaired t-test with two-tailed was used for statistical analysis. **P* < 0.05, ***P* < 0.01, ****P* < 0.001, *****P* < 0.0001, ns, not significant.


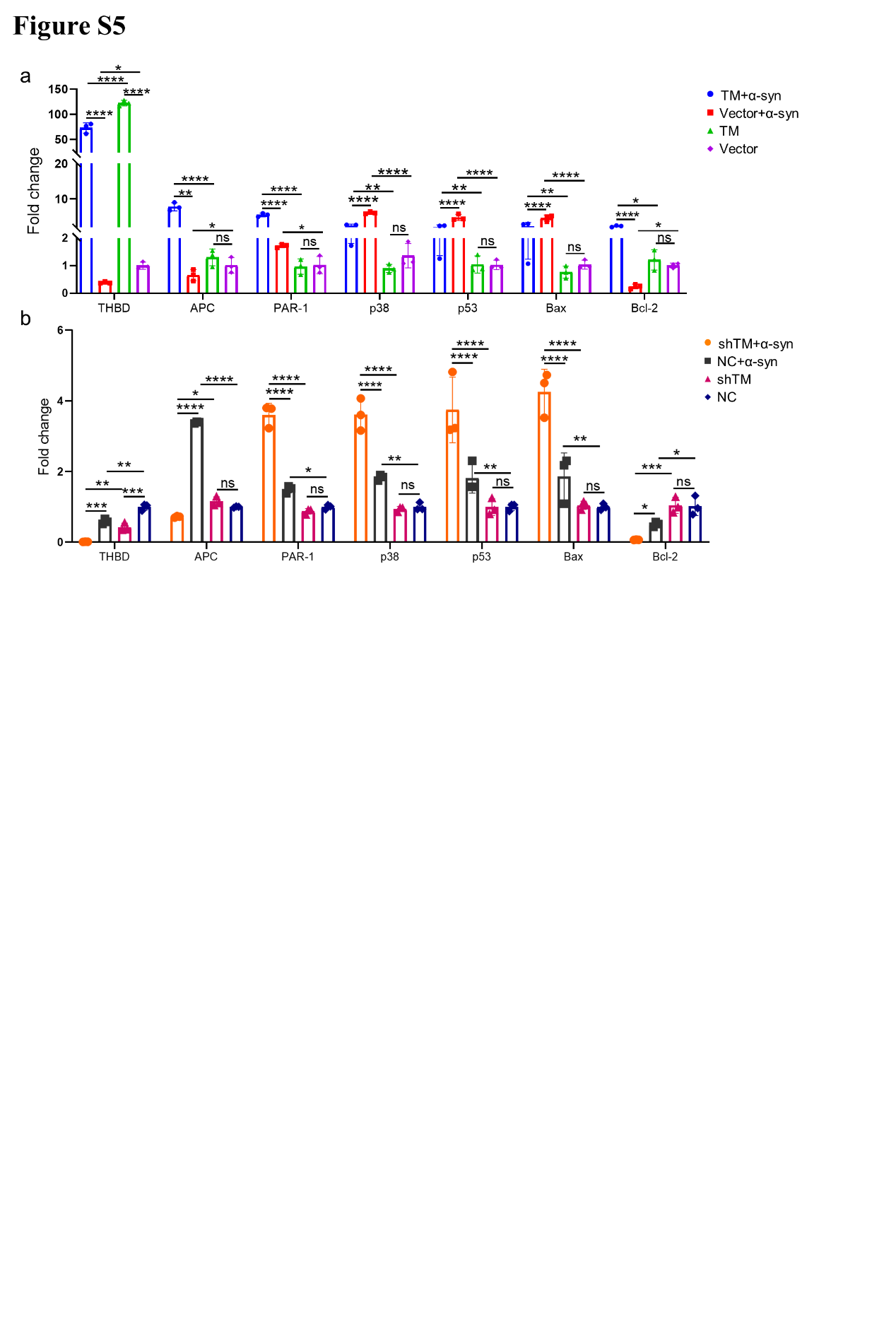


**Figure S5. TM changes mRNA levels of related proteins in apoptotic pathways. (a, b)** After infected with TM (a) or shTM (b), primary neurons were incubated with 2 μM α-syn oligomers for 48 h, the mRNA levels of THBD, APC, PAR-1, p38, p53, Bax and Bcl-2 were detected by qPCR. n = 3 represents three independent experiments. Data are mean ± SEM, and a one-way ANOVA followed by Tukey’s multiple comparison test. **P* < 0.05, ***P* < 0.01, ****P* < 0.001, *****P* < 0.0001, ns, not significant.


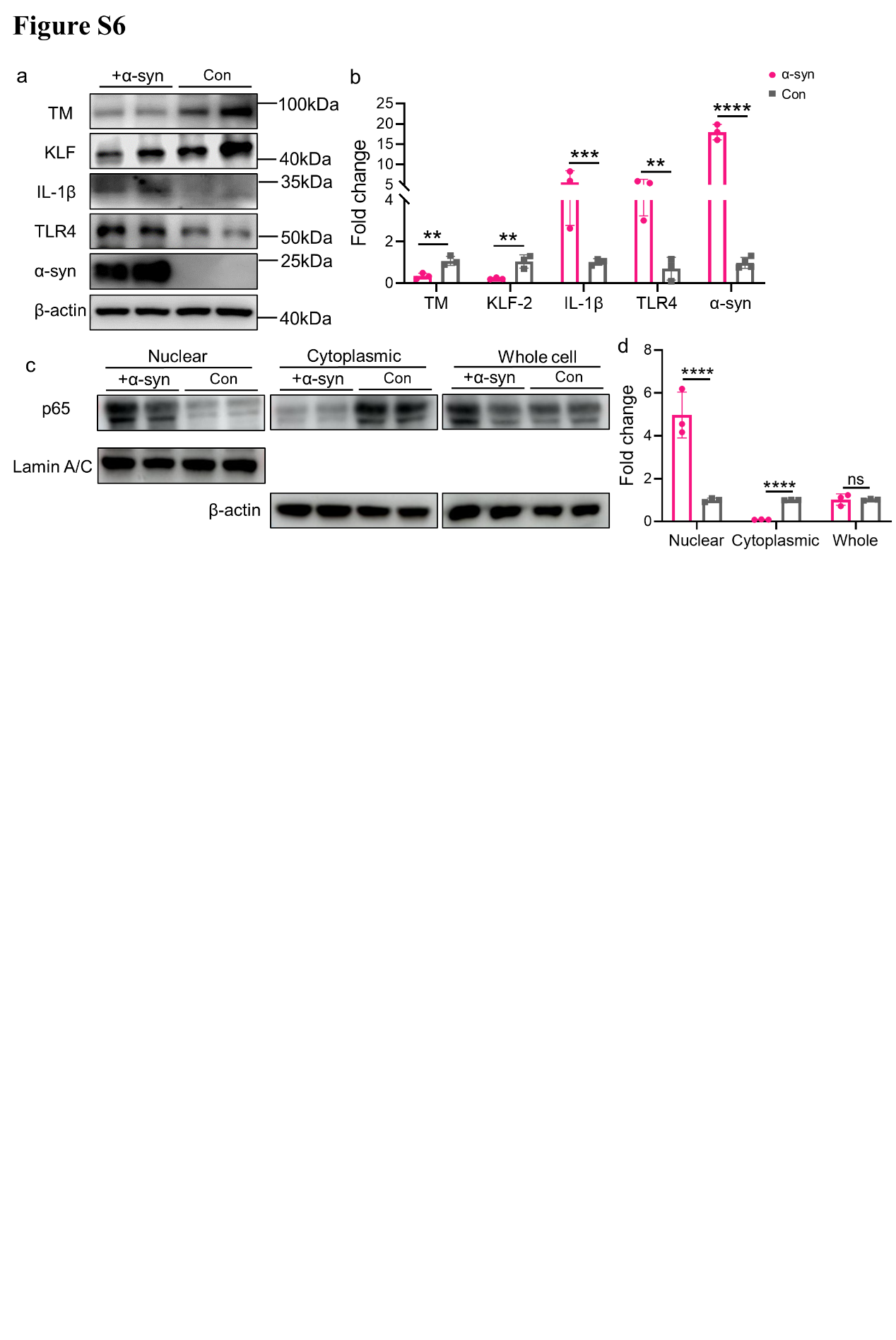


**Figure S6.** **α-syn oligomers decrease the expression of TM.** **(a)** Effects of α-syn on the levels of TM, KLF, IL-1β, and TLR4 of primary neurons. Primary neurons were incubated with 2 μM α-syn oligomers for 48 h, then the levels of TM, KLF, IL-1β, and TLR4 in primary neurons were detected by Western blotting, β-actin was used as a control. **(b)** Relative levels of TM, KLF, IL-1β, and TLR4 in (a) were quantified using Image J software, respectively. n = 3 represents three independent experiments. Data are mean ± SEM, and a one-way ANOVA followed by Tukey’s multiple comparison test. **(c)** α-syn increased the level of p65 in the nucleus. **(d)** Relative levels of p65 in (c) were quantified using Image J software, respectively. n = 3 represents three independent experiments. Data are mean ± SEM, and a one-way ANOVA followed by Tukey’s multiple comparison test. ***P* < 0.01, ****P* < 0.001, *****P* < 0.0001.


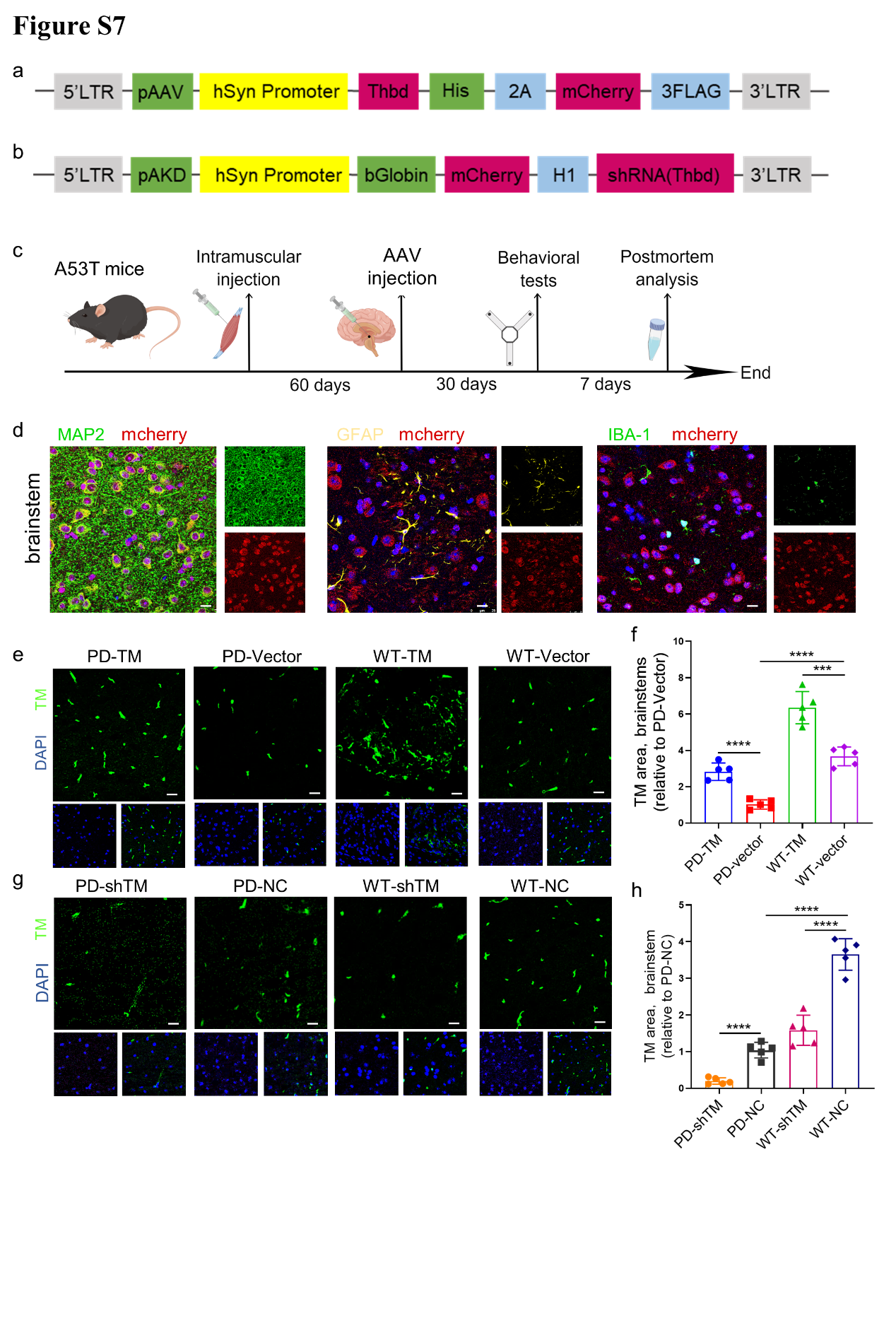


**Figure S7. The expression of TM in the brains of mice injected with TM or shTM. (a-c)** Schematic of AAV expressing mCherry and TM (a) or shTM (b) and experimental timeline (c). **(d)** Representative confocal images of mCherry^+^ cells in the brainstem of mice injected with AAV-TM (TM), AAV-Vector (vector), AAV-shTM (shTM) or AAV-NC (NC) and the co-localization of mCherry^+^ cells with MAP2, GFAP or Iba-1, respectively. Scale bar represents 5 μm. **(e, g)** Confocal micrograph of TM in the brains of A53T α-syn mice and wild-type (WT) mice injected with TM (e) or shTM (g) images of TM (green) in the brainstem. Scale bar represents 5 μm. **(f, h)** Densitometry analysis of TM in (e) or (g). n = 3. The fluorescent area of TM was quantified by Image J software. n = 5 mice per group. Data are mean ± SEM, and a one-way ANOVA followed by Tukey’s multiple comparison test was used for statistical analysis. ****P* < 0.001, *****P* < 0.0001.

**
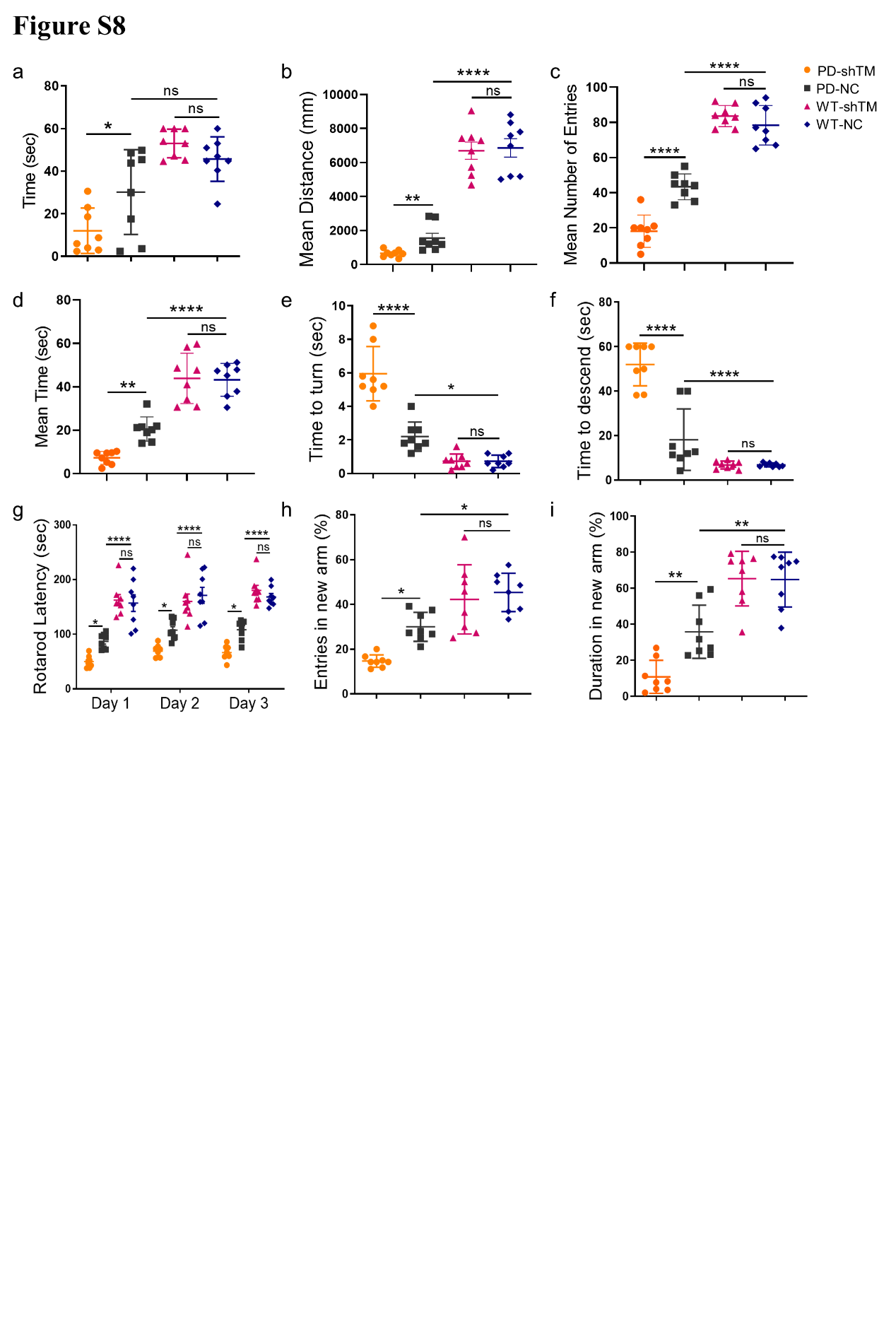
**

**Figure S8.** **TM knockdown aggravates motor defects in A53T α-syn mice.** The mice were stereotaxic injected with AAV-shTM or AAV-NC, their motor behavioral and cognitive abilities were tested 30 days after injection. **(a)** Body suspension test was used to detect mouse muscle by recording the time in which the mice hold on the rung. n = 8 mice per group. Data are mean ± SEM, and a one-way ANOVA followed by Tukey’s multiple comparison test was used for statistical analysis. **(b-d)** The motor activity of mice was detected via the distance in center (b), times to entire center region (c) and time of mice (d) spent in center. n = 8 mice per group. Data are mean ± SEM, and a one-way ANOVA followed by Tukey’s multiple comparison test was used for statistical analysis. **(e, f)** The balance and coordination of mice detected by pole test. The time of mice with shTM to turn (e) and descend (f) was recorded in this experiment. n = 8 mice per group. Data are mean ± SEM, and a one-way ANOVA followed by Tukey’s multiple comparison test was used for statistical analysis. **(g)** The exercise ability of mice was measured by the rotarod test, the latency to fall from rotarod during the test in 3 consecutive days. n = 8 mice per group. Data are mean ± SEM, and a one-way ANOVA followed by Tukey’s multiple comparison test was used for statistical analysis. **(h, i)** The special memory of mice detected by Y maze, the time spent in the novel arm (h) and the number of entries (i) in Y-maze test. n = 8 mice per group. Data are mean ± SEM, and a one-way ANOVA followed by Tukey’s multiple comparison test was used for statistical analysis. **P* < 0.05, ***P* < 0.01, *****P* < 0.0001, ns, not significant.

**
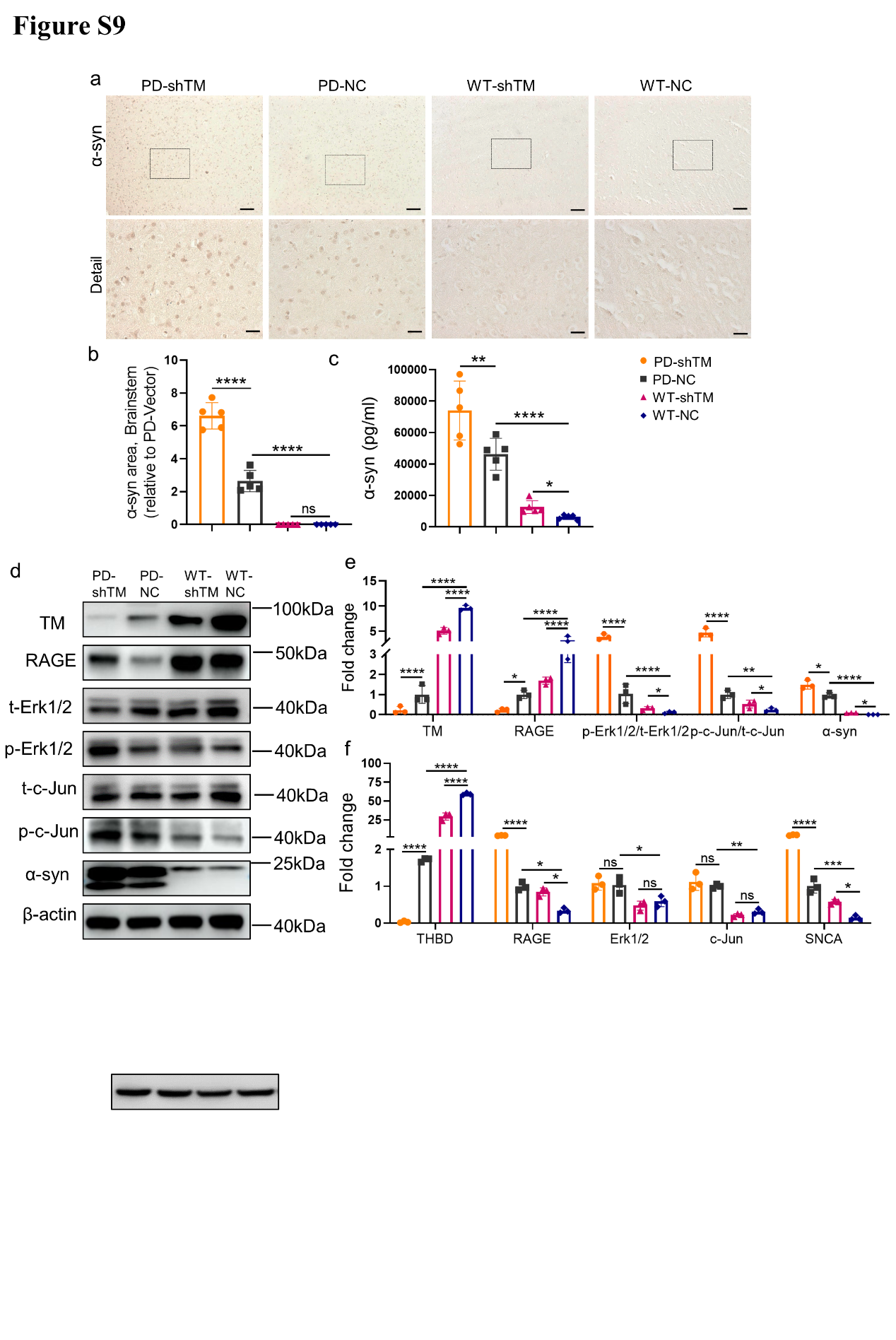
**

**Figure S9. TM Knockdown significantly** **increase α-syn and decreases the level of dopamine in the brain of A53T mice. (a)** Representative images of α-syn in brainstem of mice treated with shTM. Scale bar: 100um, Detail Scale bar: 20um. **(b)** The area of α-syn in brainstem of mouse brains in (a) was quantified using IpWin32 software. n = 5 mice per group. Data are mean ± SEM, and a one-way ANOVA followed by Tukey’s multiple comparison test was used for statistical analysis. **(c)** α-syn in the brain homogenate of mice treated with shTM were analyzed by ELISA kit. n = 5 mice per group. Data are mean ± SEM, and a one-way ANOVA followed by Tukey’s multiple comparison test was used for statistical analysis. **(d)** The levels of TM, RAGE, t-Erk1/2, p-Erk1/2, t-c-Jun, p-c-Jun and α-syn in brainstem of A53T mice with TM knockdown were analyzed by Western blotting. β-actin was used as a control. **(e)** Relative levels of TM, RAGE, t-Erk1/2, p-Erk1/2, t-c-Jun, p-c-Jun and α-syn in (d) were quantified using Image J software. n = 3 represents three independent experiments. Data are mean ± SEM, and a one-way ANOVA followed by Tukey’s multiple comparison test. **(f)** The mRNA levels of THBD, RAGE, Erk1/2, c-Jun and α-syn in the brainstem with shTM. n = 3 represents three independent experiments. Data are mean ± SEM, and a one-way ANOVA followed by Tukey’s multiple comparison test was used for statistical analysis. **P* < 0.05, ***P* < 0.01, ****P* < 0.001, *****P* < 0.0001, ns, not significant.

**
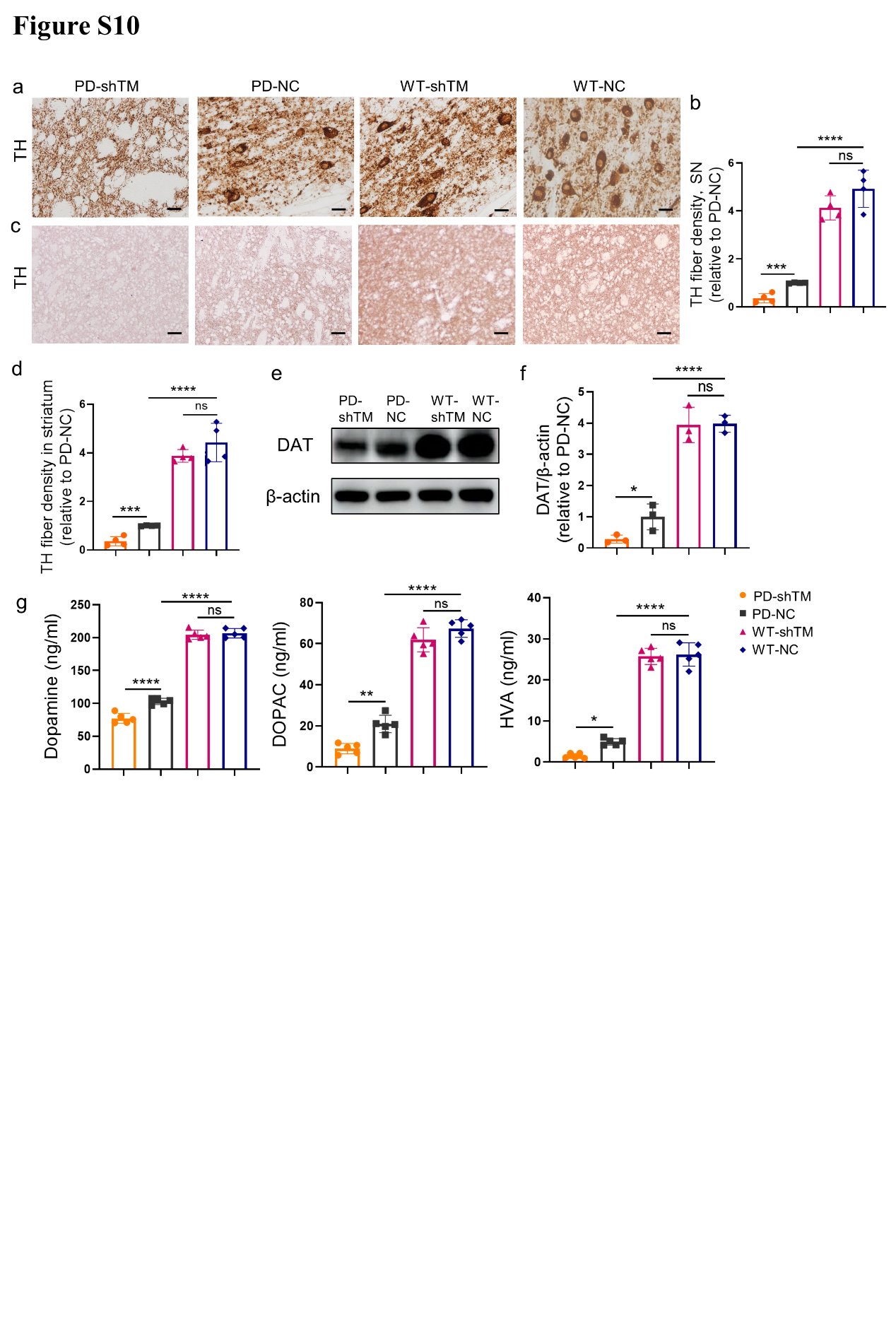
**

**Figure S10.** **TM knockdown reduces dopamine expression of in the brains of A53T mice. (a)** The levels of TH in substantia nigra of mice with shTM were detected by immunohistochemistry with anti-TH antibody. Scale bar: 20 μm. **(b)** Quantification of TH fiber densities in (a) using IpWin32 software. n = 4 mice per group. Data are mean ± SEM, and a one-way ANOVA followed by Tukey’s multiple comparison test was used for statistical analysis. **(c)** The levels of TH in striatum of mice with shTM were detected by IHC. Scale bar: 100 μm. **(d)** Quantification of TH fiber densities in (c) using IpWin32 software. n = 4 mice per group. Data are mean ± SEM, and a one-way ANOVA followed by Tukey’s multiple comparison test was used for statistical analysis. **(e)** The levels of DAT in the substantia nigra of PD mice treated with shTM were analyzed by Western blotting. β-actin was used as a control. **(f)** Densitometry analysis of DAT in (e). n = 3 represents three independent experiments. Data are mean ± SEM, and an unpaired t-test with two-tailed was used for statistical analysis. **(g)** DA and its metabolites DOPAC, HVA in the brainstem of mice treated with shTM were analyzed by ELISA kit. n = 5 mice per group. Data are mean ± SEM, and a one-way ANOVA followed by Tukey’s multiple comparison test was used for statistical analysis.

**
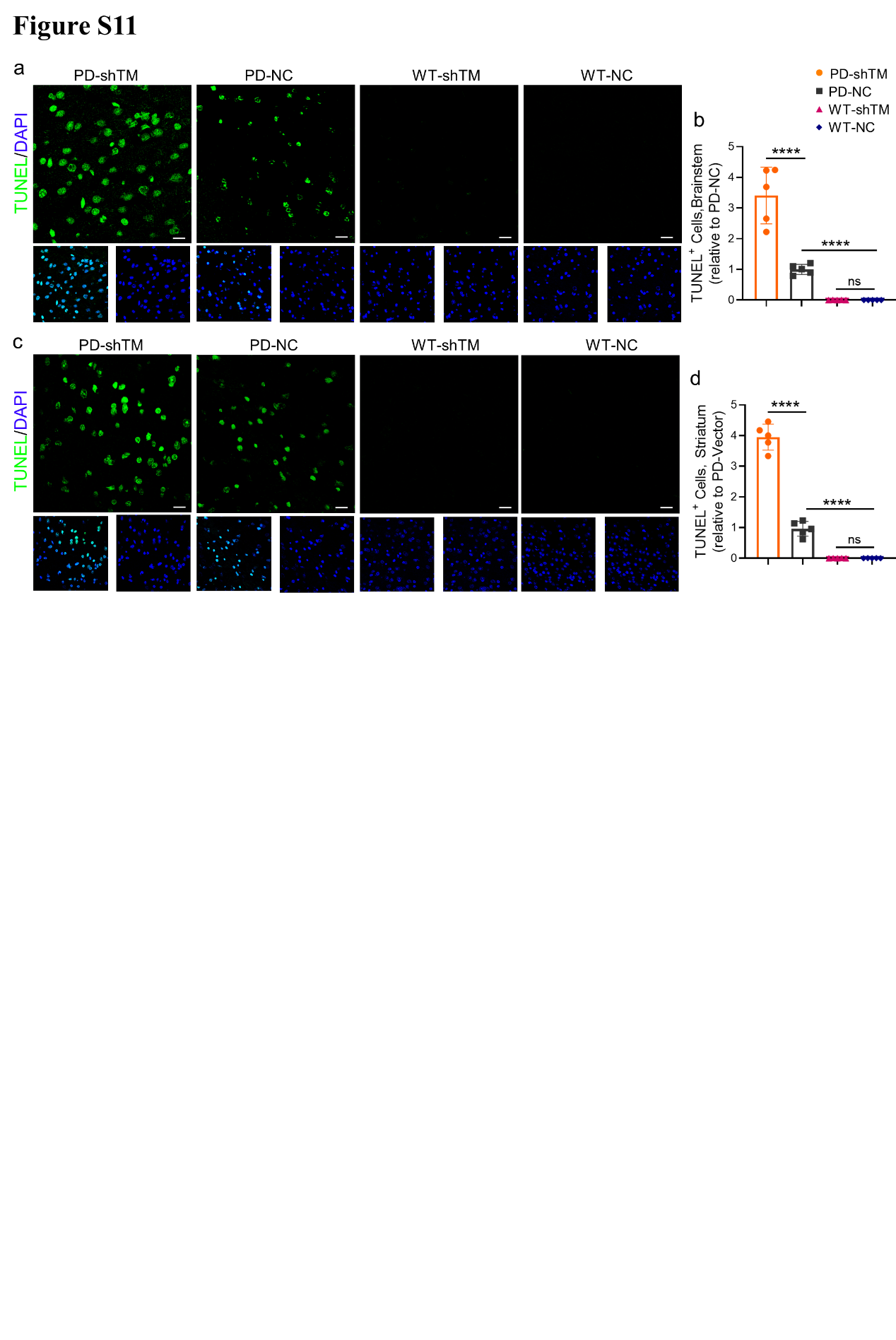
**

**Figure S11. TM affect the levels of apoptosis in the brains of A53T α-syn mice.** **(a)** Representative images of TUNEL (green) and DAPI (blue) in brainstem of A53T mice treated with shTM. Scale bar represents 20 μm. **(b)** Quantification of TUNEL^+^ cells in the brainstem regions in (a). n = 5 mice per group. Data are mean ± SEM, and a one-way ANOVA followed by Tukey’s multiple comparison test was used for statistical analysis. **(c)** Representative images of TUNEL (green) and DAPI (blue) in striatum of treated with shTM. Scale bar represents 20 μm. **(d)** Quantification of TUNEL^+^ cells in the striatum regions in (c). n = 5 mice per group. Data are mean ± SEM, and a one-way ANOVA followed by Tukey’s multiple comparison test was used for statistical analysis. *****P* < 0.0001, ns, not significant.

**
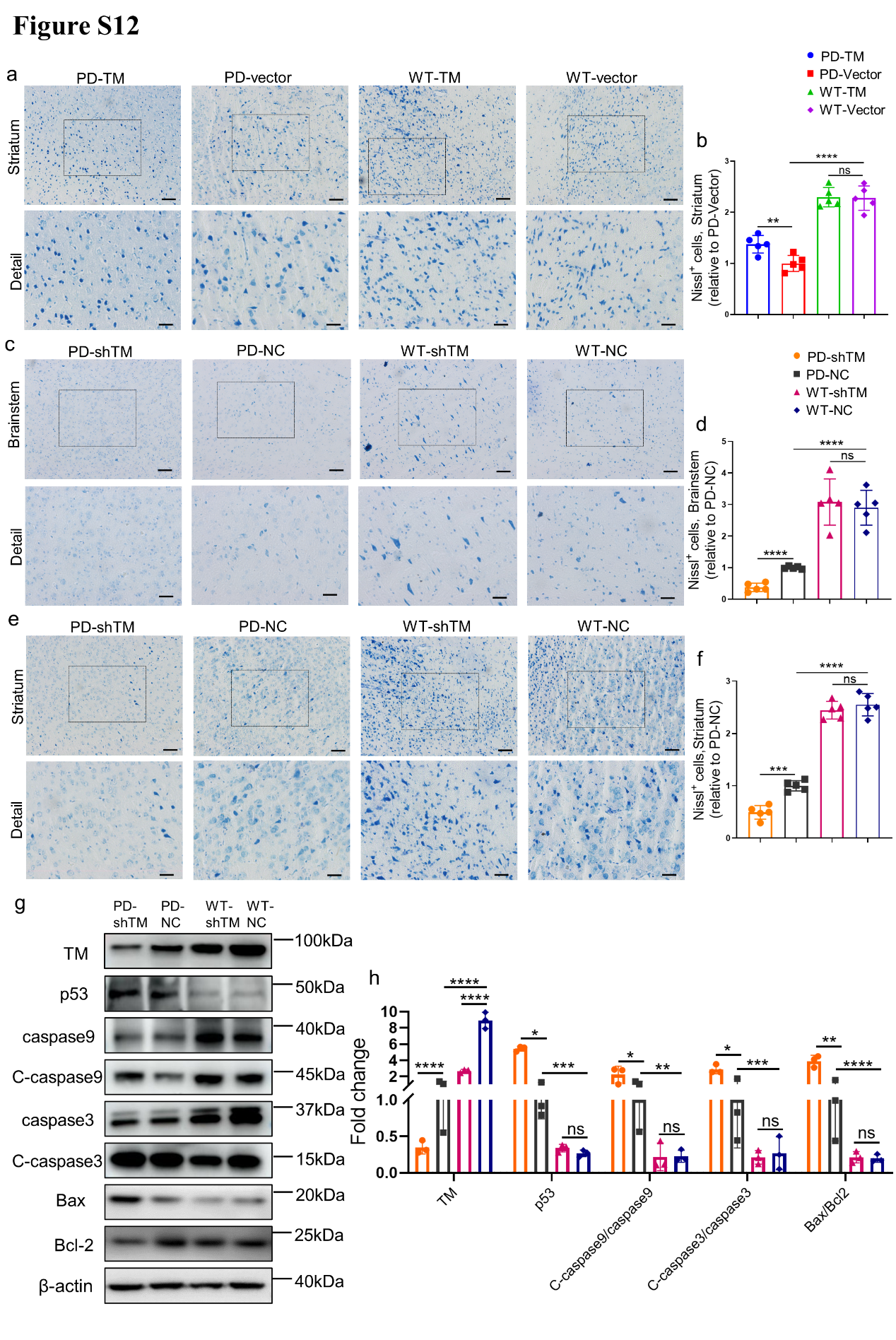
**

**Figure S12. The effects of TM on neuropathology and α-syn levels in the brains of A53T α-syn mice.** **(a)** Representative images of Nissl staining in striatum of mice treated with TM. Scale bar represents 100 μm. Detail scale bar: 50um. **(b)** Quantification of Nissl^+^ cells in striatum regions (a) using IpWin32 software. n = 5 mice per group. Data are mean ± SEM, and a one-way ANOVA followed by Tukey’s multiple comparison test was used for statistical analysis. **(c, e)** Representative images of Nissl staining in the brainstem (c) and striatum (e) of mice treated with shTM. Scale bar represents 100um. Detail scale bar: 50um. **(d, f)** Quantification of Nissl^+^ cells in brainstem regions (c) or striatum (e) using IpWin32 software. n = 5 mice per group. Data are mean ± SEM, and a one-way ANOVA followed by Tukey’s multiple comparison test was used for statistical analysis. **(g)** α-syn and apoptosis-related proteins in homogenate of shTM-treated mouse brains were analyzed by Western blotting. **(h)** Relative levels of TM, p53, Caspase9, Caspase3, Bax, Bcl-2 in (e) were quantified using Image J software. n = 3 represents three independent experiments. Data are mean ± SEM, and a one-way ANOVA followed by Tukey’s multiple comparison test was used for statistical analysis. **P* < 0.05, ***P* < 0.01, ****P* < 0.001, *****P* < 0.0001, ns, not significant.

**
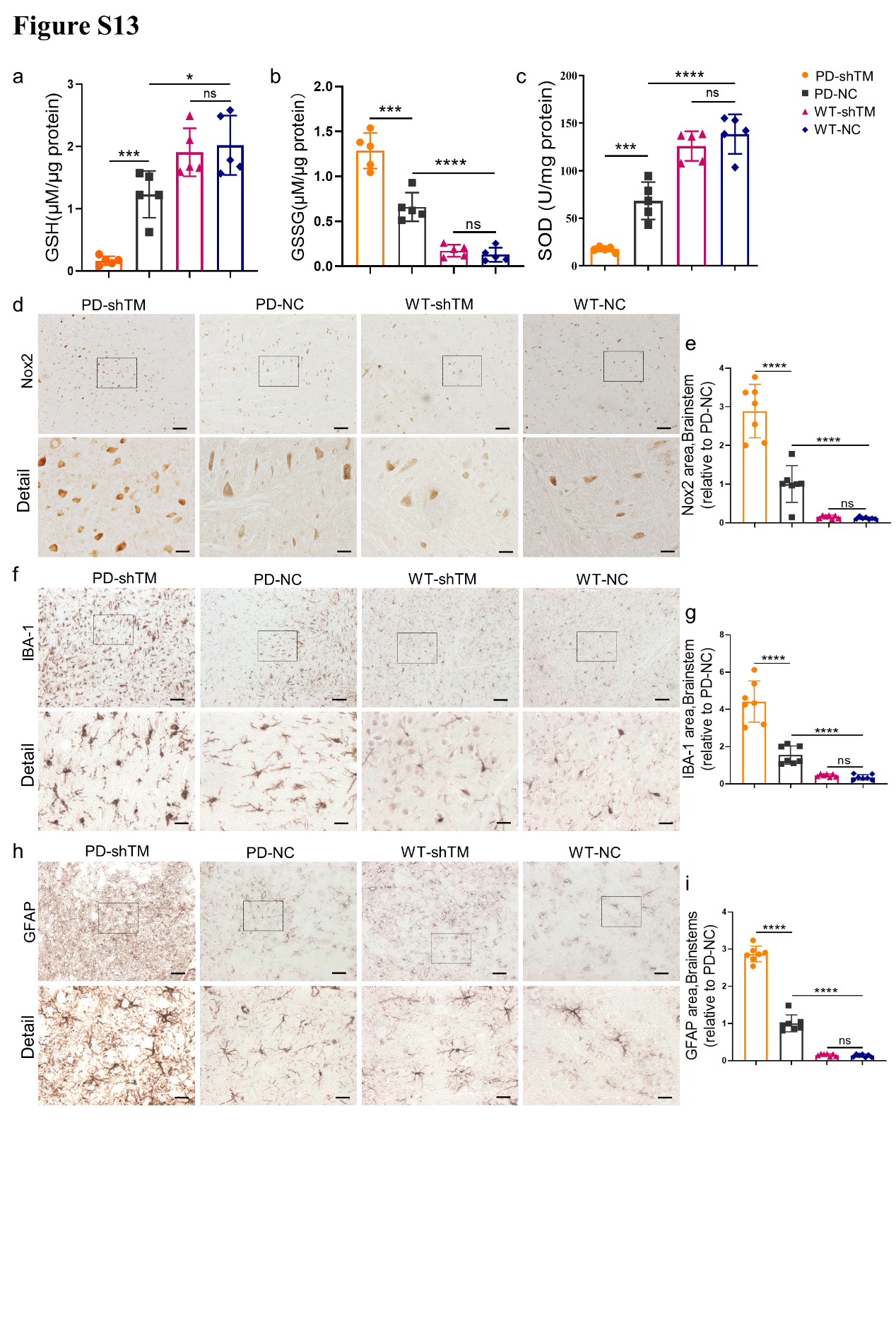
**

**Figure S13.** **TM Knockdown significantly increased the oxidative stress level of A53T α-syn mice and aggravated neuroinflammation. (a-c)** Oxidative stress related indexes GSH (a), GSSG (b) and SOD (c) in striatum of mice treated with shTM were analyzed by oxidative stress detection kit. n = 5 mice per group. Data are mean ± SEM, and a one-way ANOVA followed by Tukey’s multiple comparison test was used for statistical analysis. **(d)** Representative images of Nox2 in brainstem of mouse brains treated with shTM. Scale bar: 100um. Detail Scale bar:20um. **(e)** The area of Nox2 in brainstem of mouse brains in (d) was quantified using IpWin32 software. n = 7 mice per group. Data are mean ± SEM, and a one-way ANOVA followed by Tukey’s multiple comparison test was used for statistical analysis. **(f, h)** Representative images of microglia (f) and astrocyte (h) in brainstem of mice with shTM was detected by anti-Iba-1 and anti-GFAP antibodies, respectively. Scale bar: 100um. Detail Scale bar: 20um. **(g, i)** Quantification of the area of Iba-1 positive microglia (f) and GFAP positive astrocyte (h) in brainstem of mice by IpWin32 software. n = 7 mice per group. Data are mean ± SEM, and a one-way ANOVA followed by Tukey’s multiple comparison test was used for statistical analysis. **P* < 0.05, ****P* < 0.001, *****P* < 0.0001, ns, not significant.

**
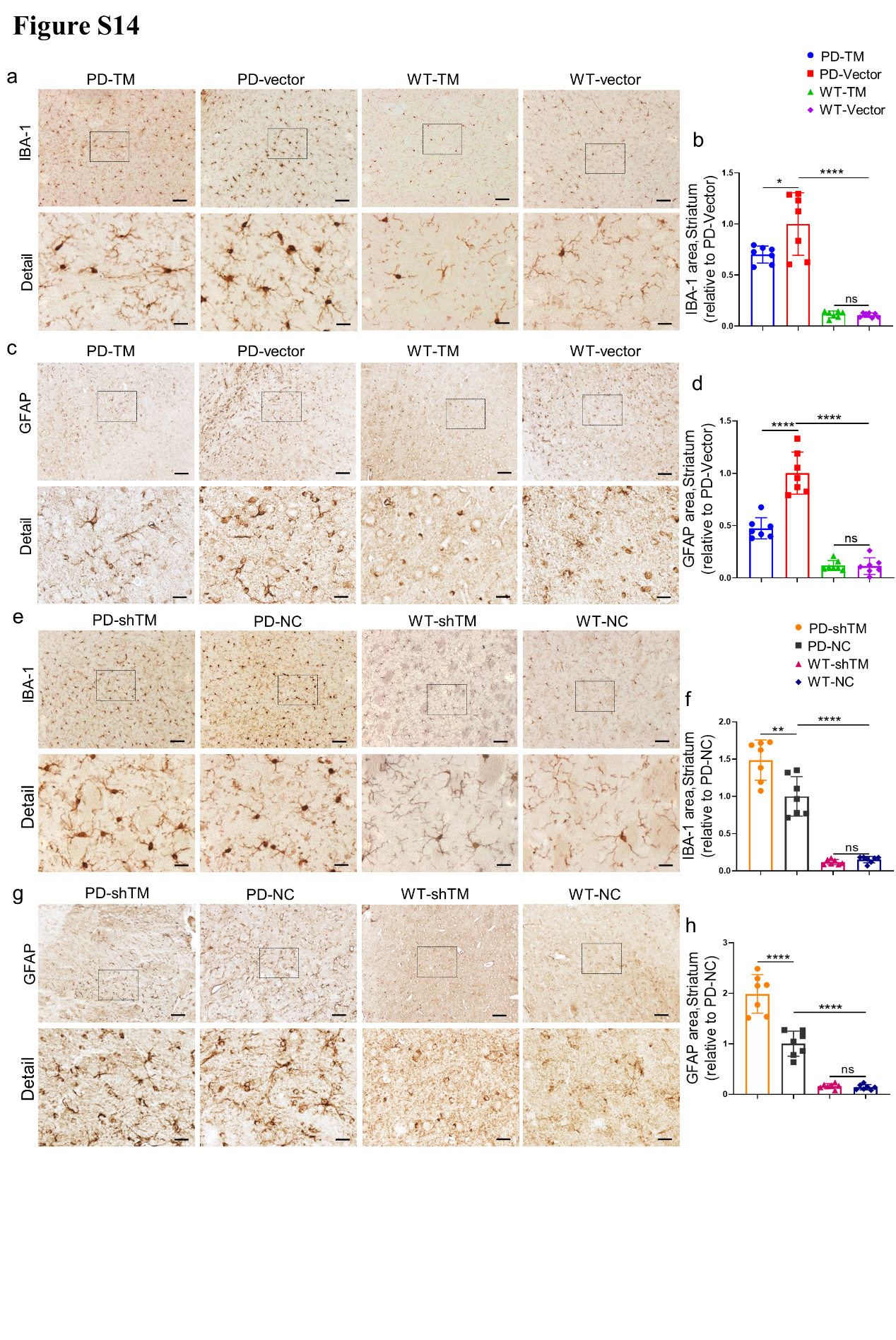
**

**Figure S14.** **TM attenuate the neuroinflammation in striatum. (a, c)** Representative images of microglia (a) and astrocytes (c) in the striatum of mouse brains with TM overexpression detected by anti-Iba-1 and anti-GFAP antibodies, respectively. Scale bar: 100um. Detail Scale bar: 20um. **(b, d)** Quantification of the area of Iba-1 positive microglia (a) and GFAP positive astrocyte (c) in striatum by IpWin32 software. n = 7 mice per group. Data are mean ± SEM, and a one-way ANOVA followed by Tukey’s multiple comparison test was used for statistical analysis. **(e, g)** Representative images of microglia (e) and astrocytes (f) in striatum of mouse brains treated with shTM detected by anti-Iba-1 and anti-GFAP antibodies, respectively. Scale bar: 100um. Detail Scale bar: 20um. **(f, h)** Quantification of the area of Iba-1 positive microglia (e) and GFAP positive astrocytes (g) in striatum by IpWin32 software. n = 7 mice per group. Data are mean ± SEM, and a one-way ANOVA followed by Tukey’s multiple comparison test was used for statistical analysis. **P* < 0.05, ***P* < 0.01, *****P* < 0.0001, ns, not significant.

**Table 1.** **Demographic details of the plasma used in this study.**

| Case | Age | Gender | Clinical diagnosis | Case | Age | Gender | Clinical diagnosis |
| --- | --- | --- | --- | --- | --- | --- | --- |
| 1 | M | 78 | PD | 24 | M | 69 | PD |
| 2 | F | 67 | PD | 25 | F | 78 | PD |
| 3 | M | 61 | PD | 26 | F | 49 | PD |
| 4 | M | 65 | PD | 27 | M | 81 | PD |
| 5 | M | 79 | PD | 28 | M | 73 | PD |
| 6 | F | 72 | PD | 29 | M | 55 | PD |
| 7 | F | 60 | PD | 30 | M | 63 | PD |
| 8 | F | 60 | PD | 31 | M | 71 | Normal |
| 9 | F | 73 | PD | 32 | F | 65 | Normal |
| 10 | F | 77 | PD | 33 | M | 55 | Normal |
| 11 | F | 70 | PD | 34 | M | 63 | Normal |
| 12 | F | 79 | PD | 35 | M | 57 | Normal |
| 13 | M | 68 | PD | 36 | M | 61 | Normal |
| 14 | M | 57 | PD | 37 | M | 59 | Normal |
| 15 | F | 70 | PD | 38 | M | 60 | Normal |
| 16 | F | 53 | PD | 39 | F | 77 | Normal |
| 17 | F | 46 | PD | 40 | F | 72 | Normal |
| 18 | M | 74 | PD | 41 | F | 66 | Normal |
| 19 | F | 69 | PD | 42 | F | 63 | Normal |
| 20 | F | 62 | PD | 43 | M | 57 | Normal |
| 21 | F | 67 | PD | 44 | M | 64 | Normal |
| 22 | M | 68 | PD | 45 | M | 66 | Normal |
| 23 | M | 75 | PD |  |  |  |  |

**PD, Parkinson’s disease; M, male; F, female.**
