# Supplementary material for: Thrombomodulin reduces α-synuclein generation and ameliorates neuropathology in a mouse model of Parkinson’s disease
Source: Cell Death Discov. 2024 Apr 8;10:167. doi: 10.1038/s41420-024-01939-y (PMC11002034; doi:10.1038/s41420-024-01939-y)
Supplement: Supplementary file 2 — Original Data [file 41420_2024_1939_MOESM2_ESM.pdf]

Figure 1

a

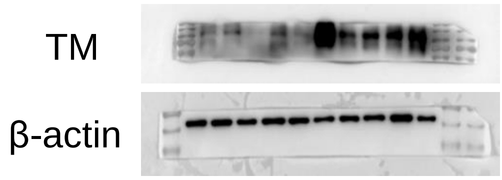

Figure 2

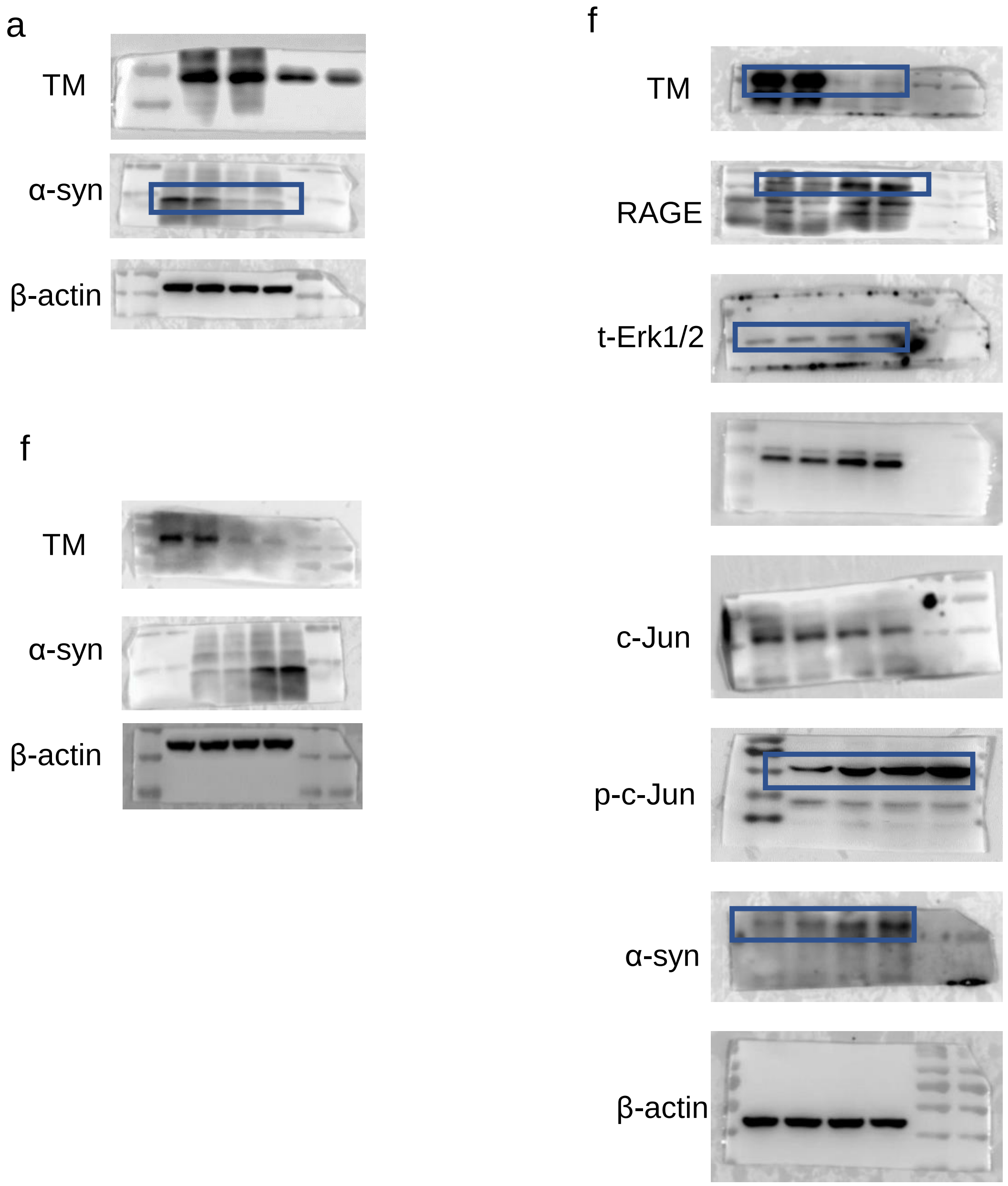
a
TM
α-syn
β-actin
f
TM
α-syn
β-actin
f
TM
RAGE
t-Erk1/2
c-Jun
p-c-Jun
α-syn
β-actin

Figure 3

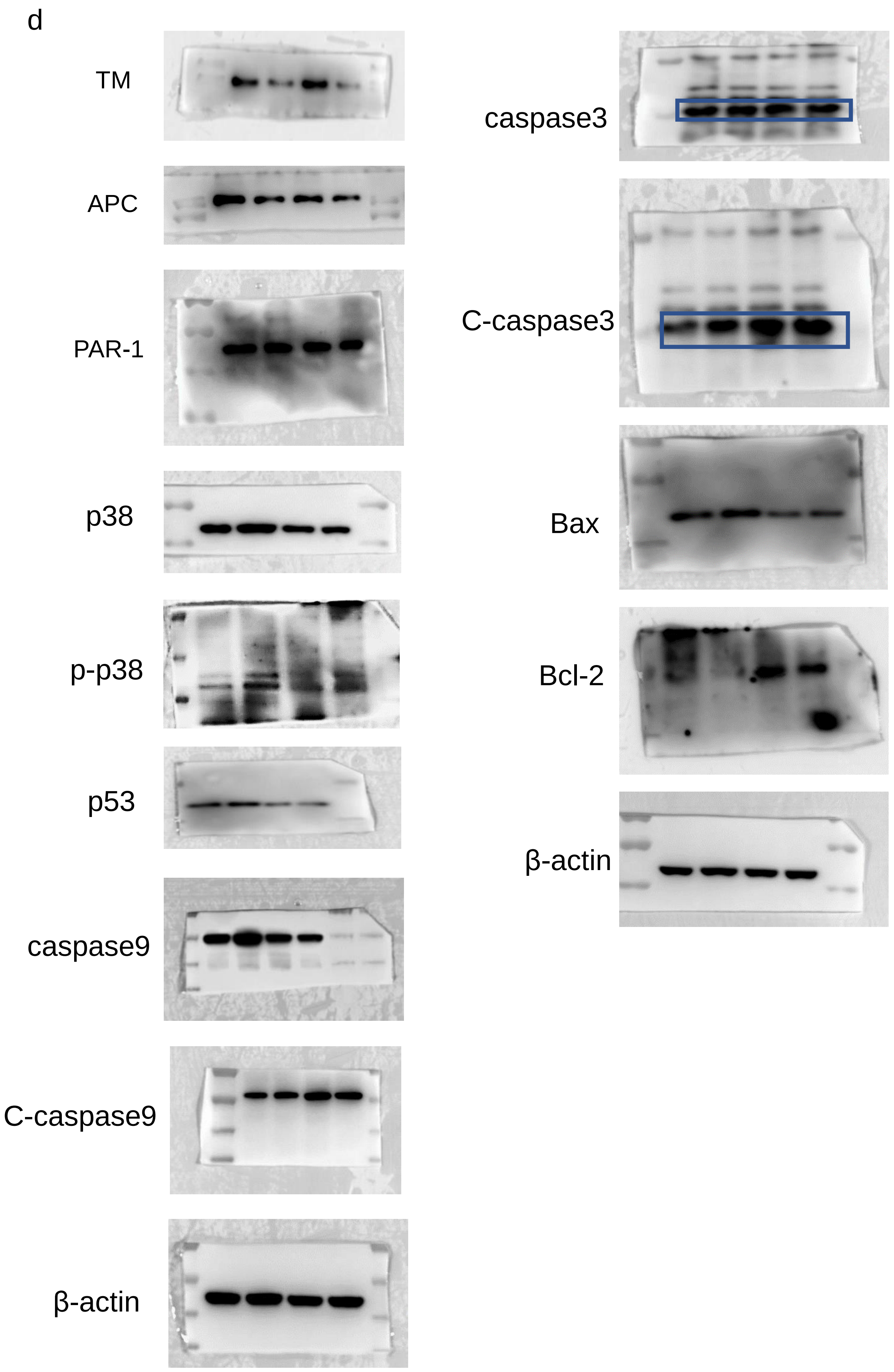
d
TM
APC
PAR-1
p38
p-p38
p53
caspase9
C-caspase9
β-actin
caspase3
C-caspase3
Bax
Bcl-2
β-actin

Figure 5

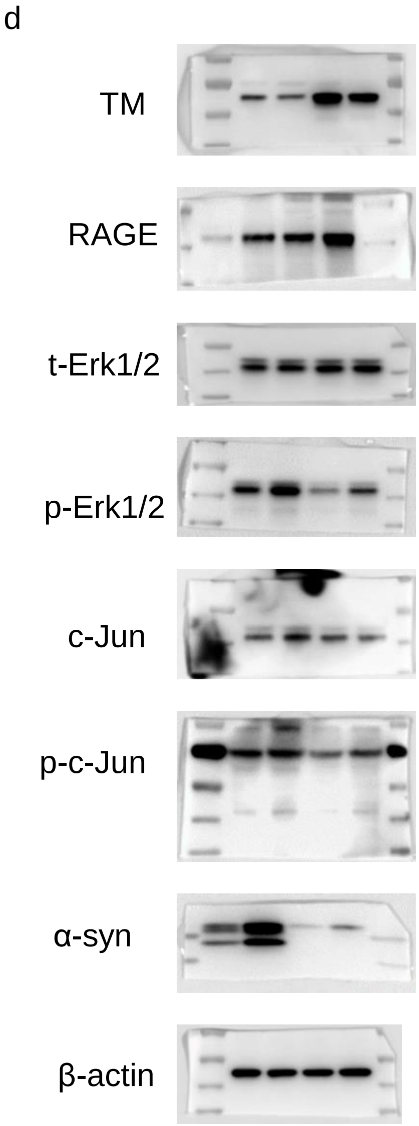

Figure 6

e

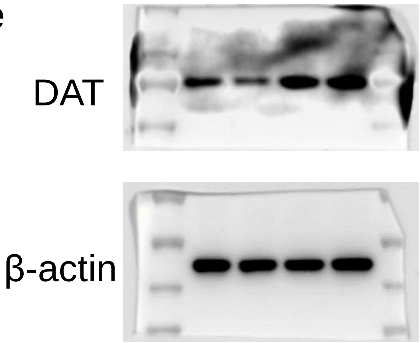

Figure 7

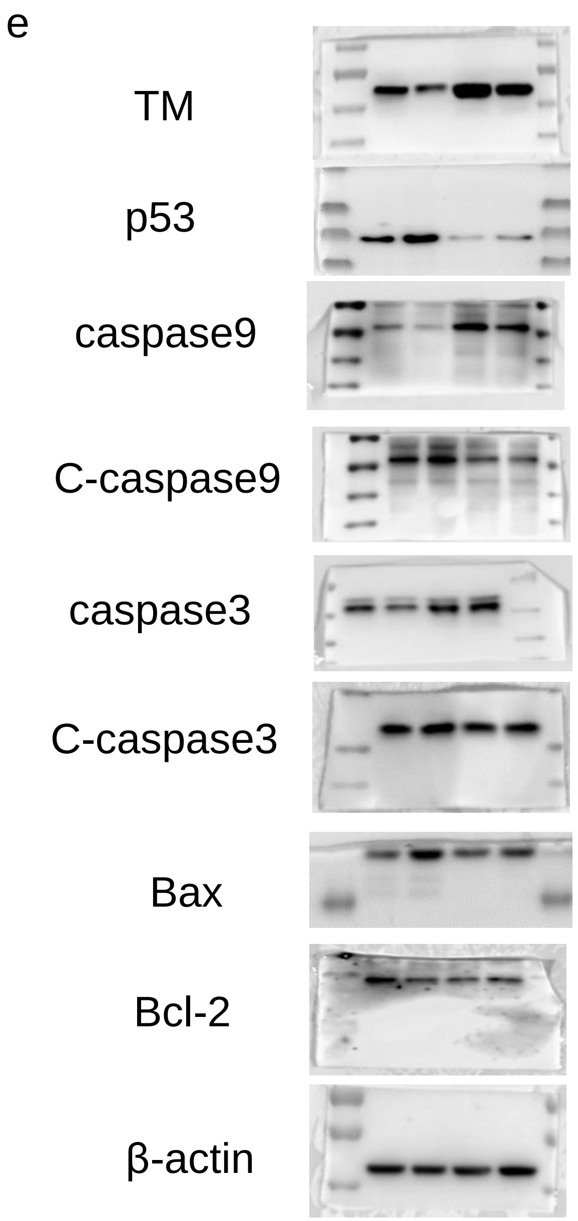
e
TM
p53
caspase9
C-caspase9
caspase3
C-caspase3
Bax
Bcl-2
β-actin

Figure S1

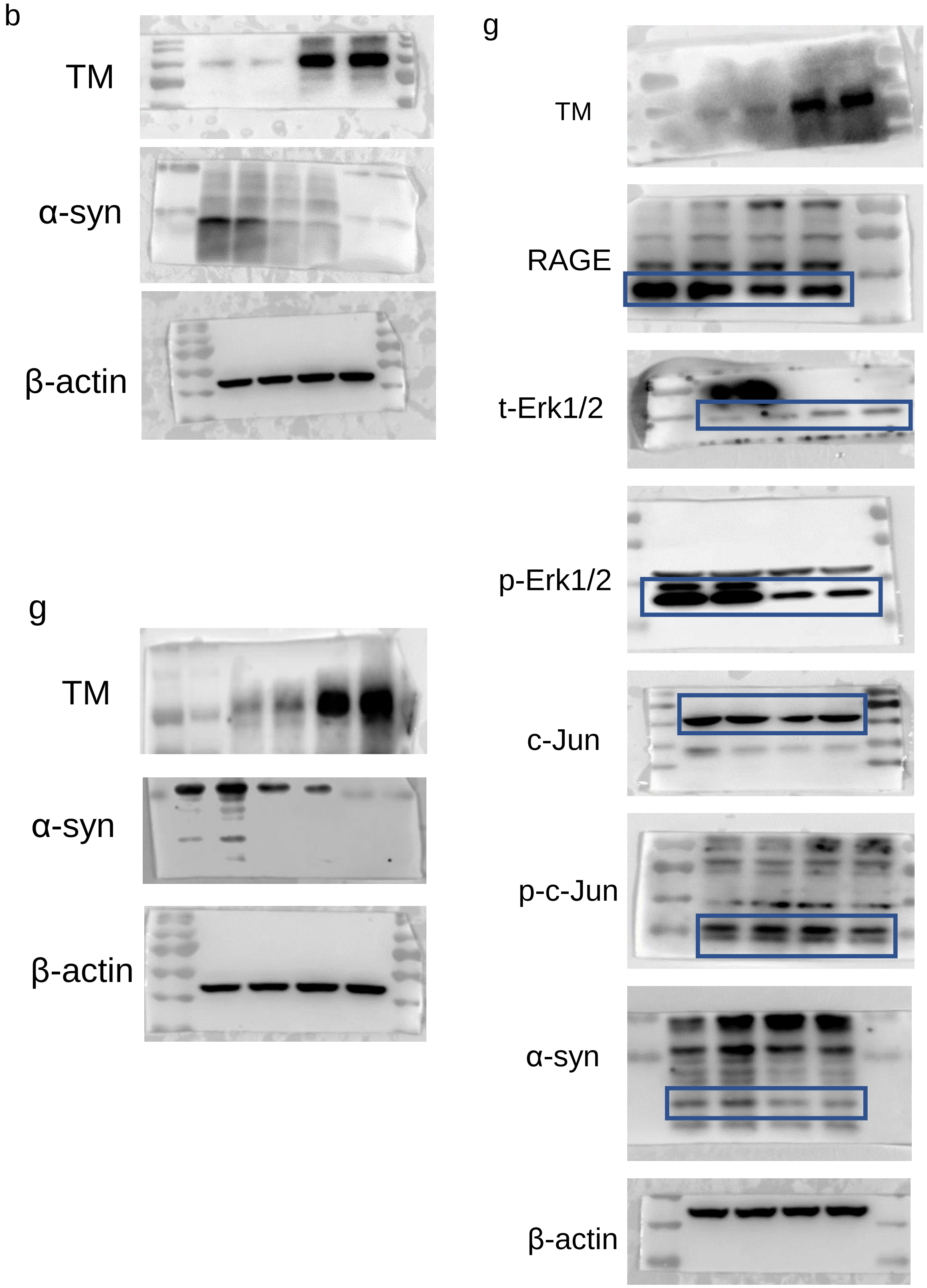

b
TM
α-syn
β-actin
g
TM
α-syn
β-actin
g
TM
RAGE
t-Erk1/2
p-Erk1/2
c-Jun
p-c-Jun
α-syn
β-actin

Figure S2

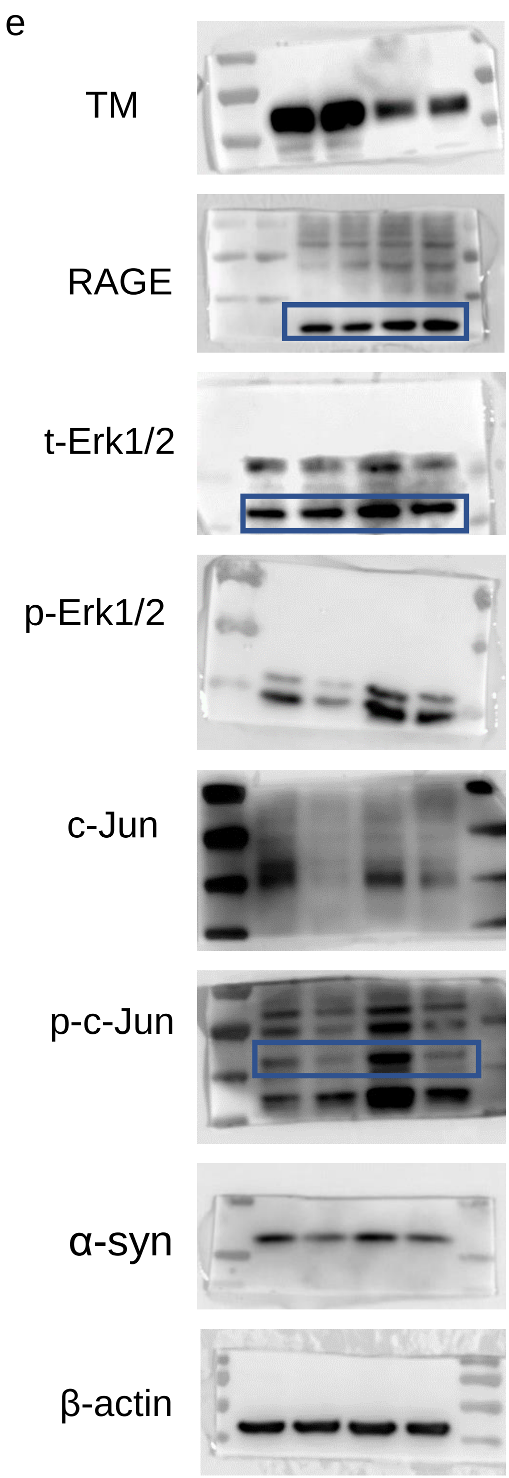

Figure S3

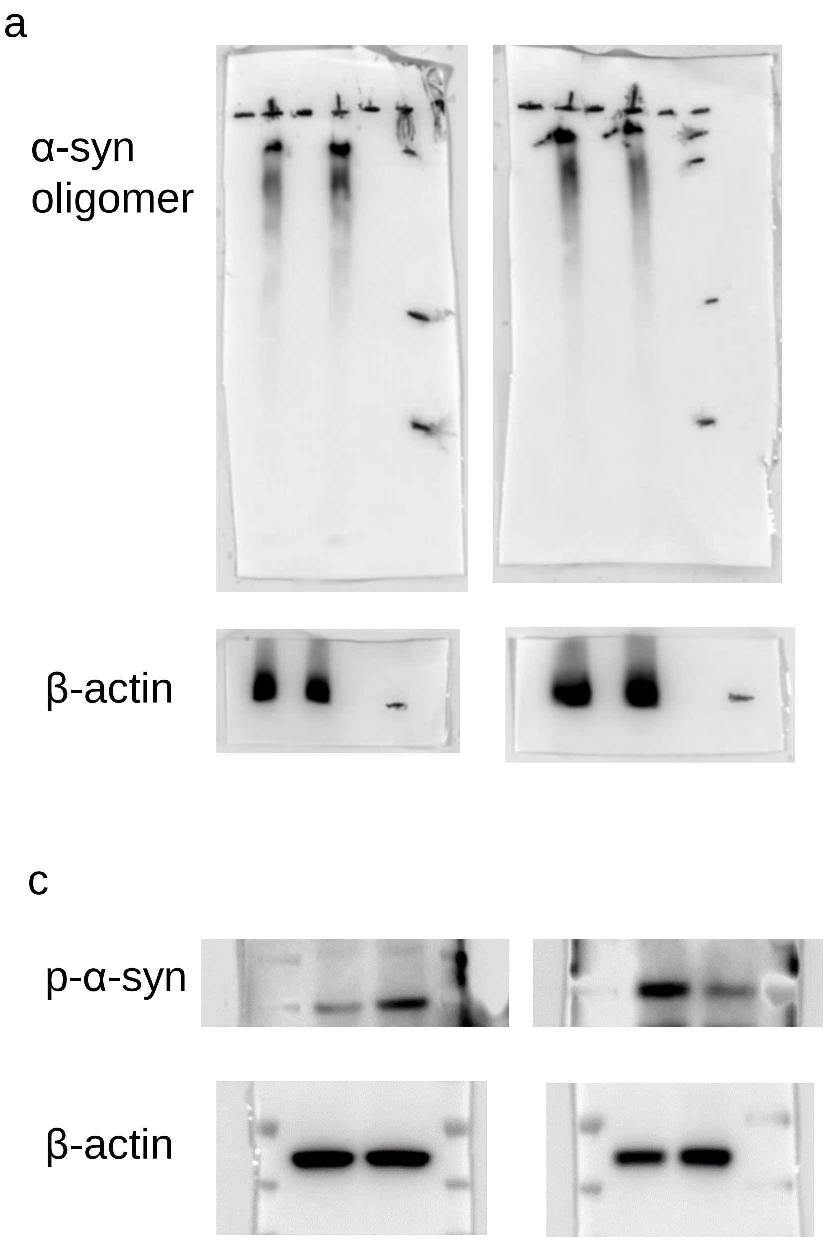

Figure S4

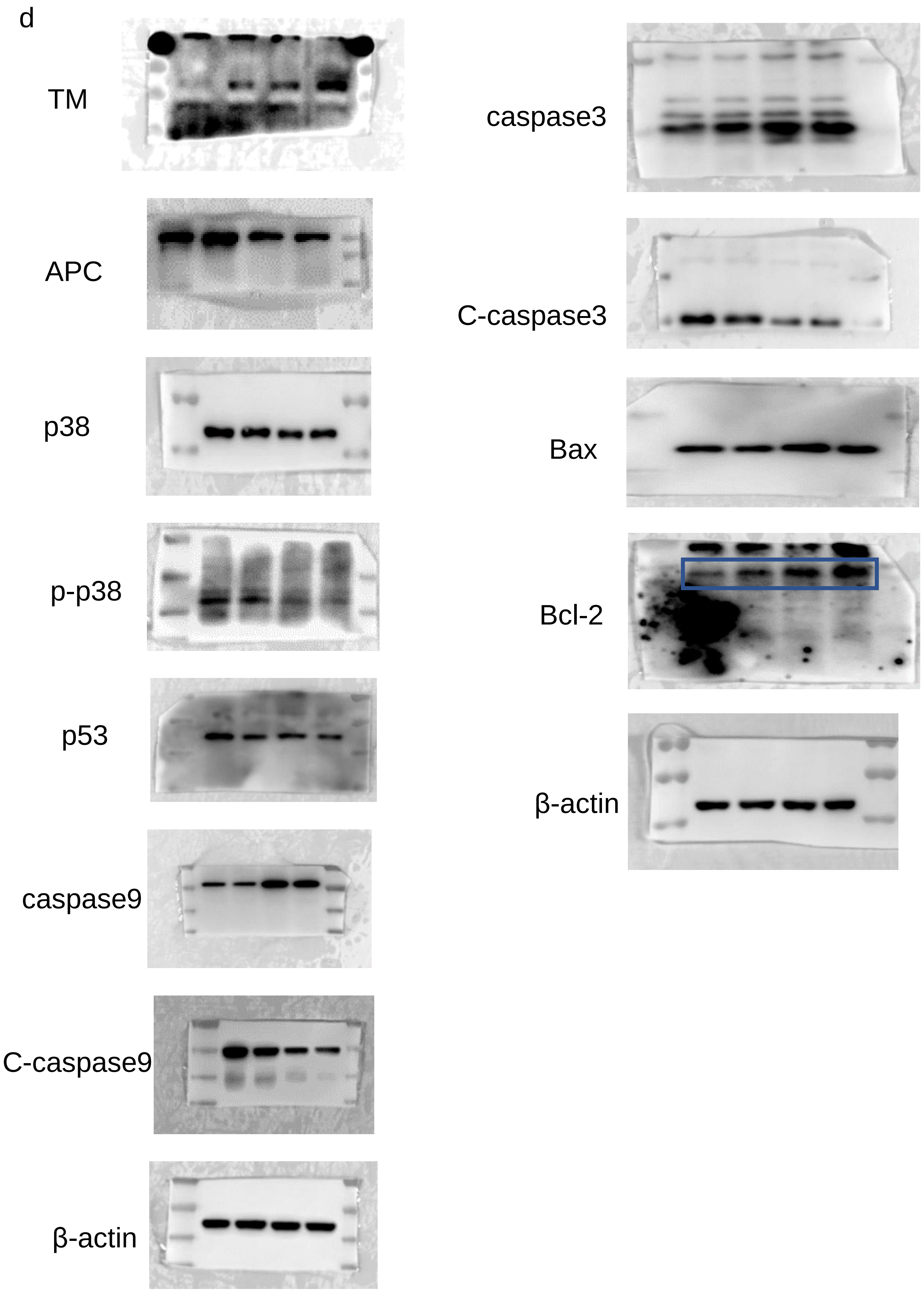
d
TM
APC
p38
p-p38
p53
caspase9
C-caspase9
β-actin
caspase3
C-caspase3
Bax
Bcl-2
β-actin

Figure S6

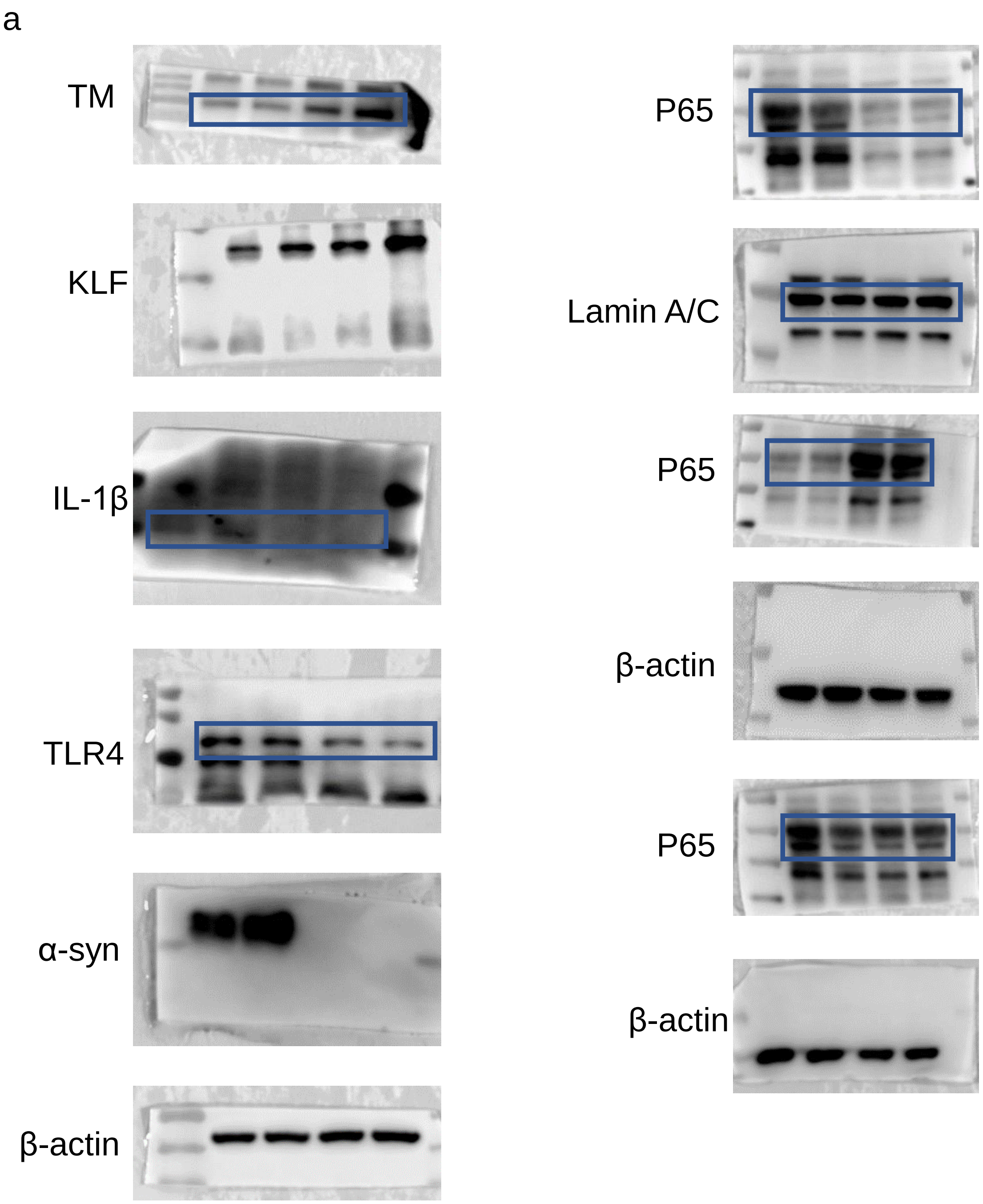
a
TM
KLF
IL-1β
TLR4
α-syn
β-actin
P65
Lamin A/C
P65
β-actin
P65
β-actin

Figure S9

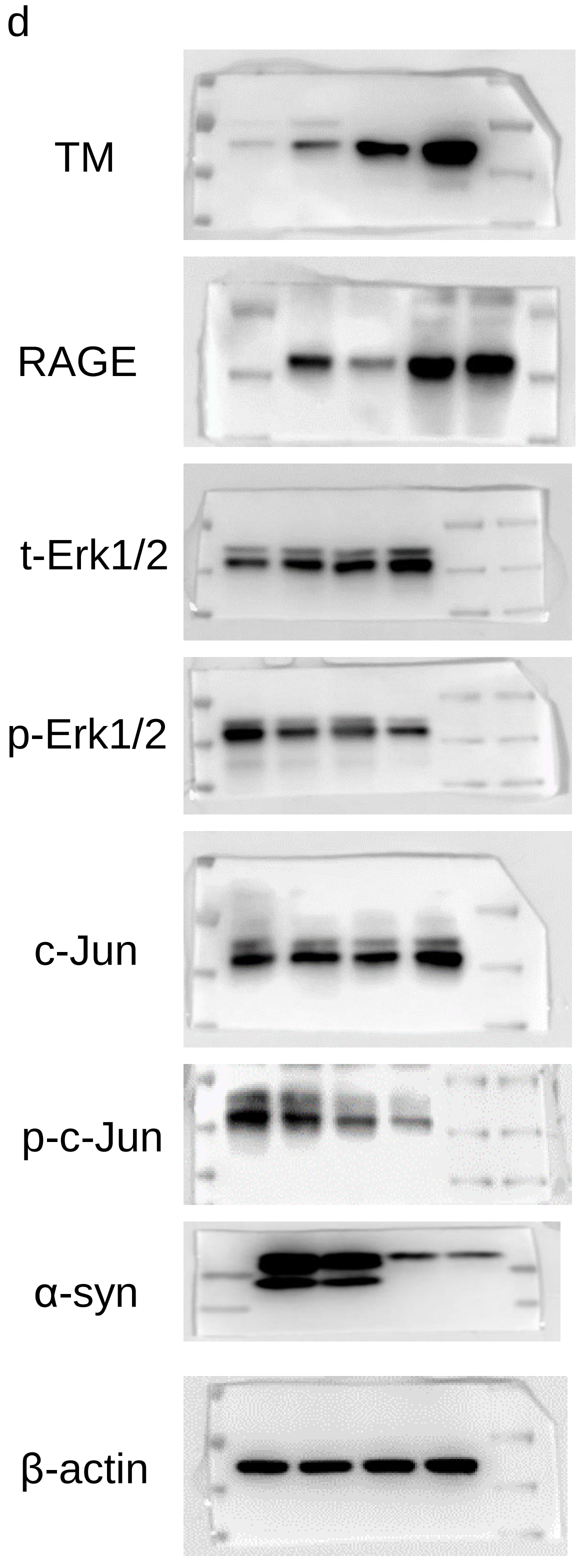

Figure S10

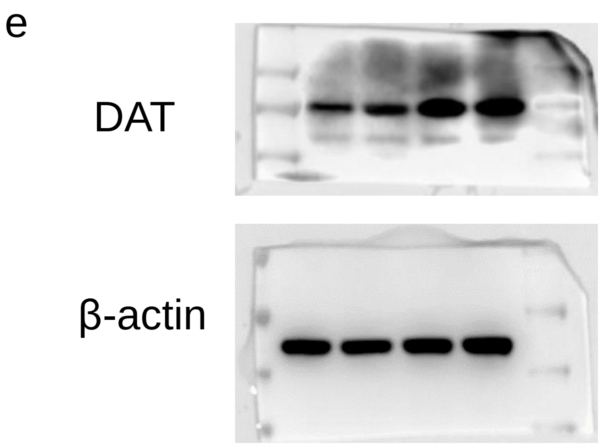

Figure S12

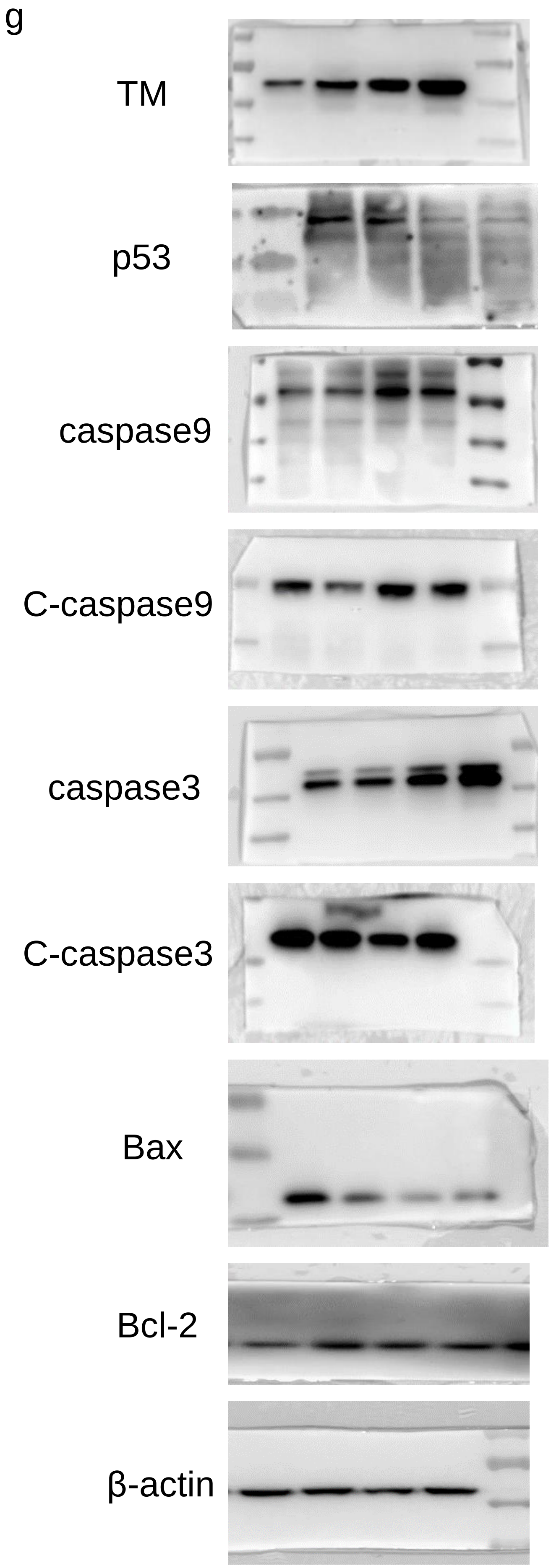
g
TM
p53
caspase9
C-caspase9
caspase3
C-caspase3
Bax
Bcl-2
β-actin
